# Engineering Synthetic Signaling Pathways with Programmable dCas9-Based Chimeric Receptors

**DOI:** 10.1016/j.celrep.2017.08.044

**Published:** 2017-09-12

**Authors:** Toni A. Baeumler, Ahmed Ashour Ahmed, Tudor A. Fulga

**Affiliations:** 1 Weatherall Institute of Molecular Medicine, Radcliffe Department of Medicine, University of Oxford, Oxford OX3 9DS, UK; 2 Ovarian Cancer Cell Laboratory, Weatherall Institute of Molecular Medicine, Nuffield Department of Obstetrics and Gynaecology, University of Oxford, Oxford OX3 9DU, UK

**Keywords:** synthetic receptors, chimeric receptors, CRISPR, dCas9-VP64, split Cas9, GPCR, RTK, genome engineering, dCas9-synR, transcriptional programs

## Abstract

Synthetic receptors provide a powerful experimental tool for generation of designer cells capable of monitoring the environment, sensing specific input signals, and executing diverse custom response programs. To advance the promise of cellular engineering, we have developed a class of chimeric receptors that integrate a highly programmable and portable nuclease-deficient CRISPR/Cas9 (dCas9) signal transduction module. We demonstrate that the core dCas9 synthetic receptor (dCas9-synR) architecture can be readily adapted to various classes of native ectodomain scaffolds, linking their natural inputs with orthogonal output functions. Importantly, these receptors achieved stringent OFF/ON state transition characteristics, showed agonist-mediated dose-dependent activation, and could be programmed to couple specific disease markers with diverse, therapeutically relevant multi-gene expression circuits. The modular dCas9-synR platform developed here provides a generalizable blueprint for designing next generations of synthetic receptors, which will enable the implementation of highly complex combinatorial functions in cellular engineering.

## Highlights

• dCas9-synRs based on a highly programmable split-dCas9 signal transduction module
• Core architecture can be standardized across multiple classes of sensing domains
• Integration of user-controlled AND gates in the basic dCas9-synR architecture
• dCas9-synRs enable implementation of complex combinatorial therapeutic responses

## Graphical Abstract

**Figure:**
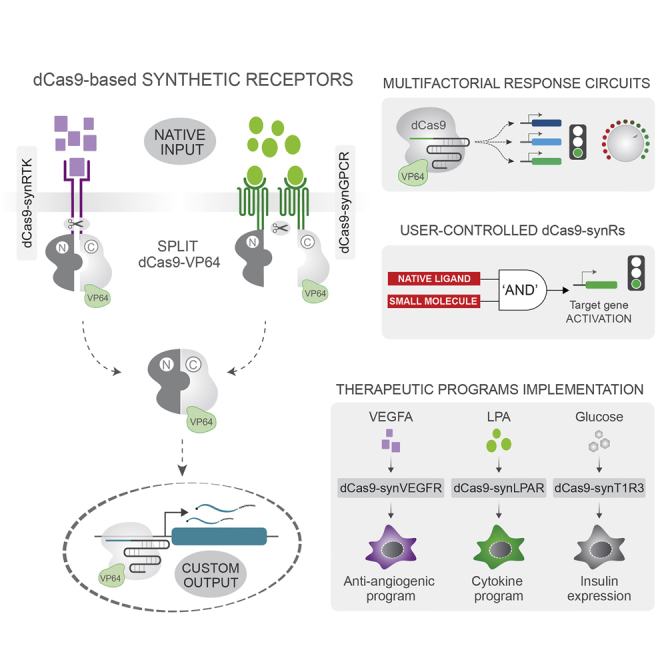

## Teaser

Using a highly programmable split-dCas9-based signal transduction module, Baeumler et al. have created a novel class of synthetic receptors (dCas9-synRs) capable of coupling biologically relevant input signals with the direct activation of custom user-defined output response programs. dCas9-synRs expand the promise of cellular engineering for research and therapeutic applications.

## Introduction

Signal integration and transduction by cell-surface receptors is a complex, multi-layered process leading to allosteric activation of downstream mediators, which in turn elicit a predefined cellular response (Lim et al., 2014). The modular architecture of most transmembrane receptors provides a unique opportunity for engineering novel sensor/effector circuits, enabling the evolution of custom cellular functions for research and therapeutic applications (Lienert et al., 2014, Lim, 2010, Lim and June, 2017). By modifying either the input-sensing ectodomains or the intracellular signaling modules, rationally designed programmable synthetic receptors can be used to assemble unconventional signaling cascades orthogonal to endogenous pathways. So far, the design of such chimeric receptors relied mainly on two basic conceptual frameworks: (1) coupling synthetic (or altered) ligand-binding domains with native signal transduction modules (Conklin et al., 2008), or (2) fusing native or engineered ligand-sensing ectodomains with artificial transcription factors (Barnea et al., 2008, Morsut et al., 2016).

Perhaps one of the most remarkable implementations of the first strategy is the development of chimeric antigen receptors (CARs) (Gill and June, 2015, Kershaw et al., 2013, Lim and June, 2017). In general, CAR designs rely on coupling an extracellular antibody single-chain variable fragment (scFv) recognizing a cancer-specific antigen with the native intracellular signaling unit of a T cell receptor (TCR), via a transmembrane (TM) domain (Kershaw et al., 2013, Srivastava and Riddell, 2015). Importantly, transgenic expression of CARs has been successfully used to establish adoptive T cell immunotherapies targeting various forms of hematological cancers (Gill and June, 2015, Grupp et al., 2013, Turtle et al., 2016). An elegant integration of both user-specified sensing and signaling domains has been recently reported for engineering synthetic Notch receptors (synNotch) (Morsut et al., 2016). These chimeric receptors consist of customized scFv or nanobody extracellular domains, the minimal Notch transmembrane core activation mechanism, and artificial transcription factor endodomains. This versatile modular receptor architecture was adapted to respond to numerous membrane bound endogenous and synthetic ligands and drive the expression of a range of user-defined transgenes in various cell types, including primary human T cells (Morsut et al., 2016, Roybal et al., 2016a, Roybal et al., 2016b).

Although advances in receptor engineering have significantly expanded our ability to program novel cellular functions, their diversification is restricted by a relatively limited number of response modules. In the majority of current chimeric receptor paradigms, signal transduction is mediated either by endogenous intracellular modules from orthogonal receptors or by effectors fused to predefined DNA binding domains (Lienert et al., 2014, Lim, 2010, Lim and June, 2017). Therefore, most of these synthetic receptors can only activate native signaling pathways or drive the expression of pre-integrated transgenes. A self-contained modular receptor design capable of directly and precisely engaging any endogenous gene circuit would vastly expand the promise of cellular engineering and simplify the implementation of complex synthetic signaling cascades.

The nuclease-deficient type-II CRISPR-associated Cas9 protein (dCas9) has emerged as a uniquely versatile molecular scaffold for the assembly of synthetic effector proteins including programmable transcription factors (TF) (Dominguez et al., 2016, Jusiak et al., 2016). The first integration of a dCas9-TF signal transduction module in the design of synthetic receptors has been recently reported using the modular extracellular sensor architecture (MESA) technology (Schwarz et al., 2017). Although this study demonstrated the potential of engineering novel cellular functions, MESA receptors displayed significant ligand-independent activation and relatively modest agonist-mediated induction. The utility of artificial signaling pathways for cellular reprogramming largely depends on reaching optimal OFF/ON state-transition characteristics for all system components. Consequently, by analogy to native receptors, a critical consideration when engineering chimeric receptors is attaining minimal baseline activity in the absence of a cognate ligand and eliciting a robust cellular response upon agonist stimulation.

Here, we report a modular receptor framework leveraging the evolutionarily optimized ligand-sensing capacity of native receptors and the programmability of dCas9 transcription factors. The resulting synthetic dCas9-based receptors (dCas9-synR) can in principle integrate a broad variety of input signals (soluble proteins, peptides, lipids, sugars, small molecules) and regulate any cellular pathway by simply defining the dCas9-associated single guide RNAs (sgRNAs). Using an iterative optimization approach, we have engineered dCas9-synRs that display minimal OFF-state baseline activation and robust ON-state ligand-induced signal transduction. To demonstrate that the ensuing core architecture and signal release mechanism can be standardized across multiple classes of sensing domains, we created prototype RTK-based (dCas9-synRTK) and GPCR-based (dCas9-synGPCR) chimeric receptors. We show that both classes of synthetic receptors can couple a variety of soluble inputs with highly specific and robust induction of target genes in an agonist dose-dependent manner. In addition, we demonstrate parallel ligand-mediated activation of multiple endogenous genes and provide strategies for multifactorial “AND gate” control of custom cellular responses. Finally, we demonstrate that synthetic dCas9-synRs can be programmed to drive therapeutically relevant gene expression circuits in the presence of various classes of input signals including proteins, lipids, and sugars. The performance of dCas9-synR receptors and their unique versatility in redirecting cellular information flow makes them ideally suited for engineering designer therapeutic cells capable of sensing specific disease markers, and in turn, driving custom transcriptional programs.

## Results

### dCas9-TF Membrane Tethering and Protease-Mediated Release

**Figure 1:**
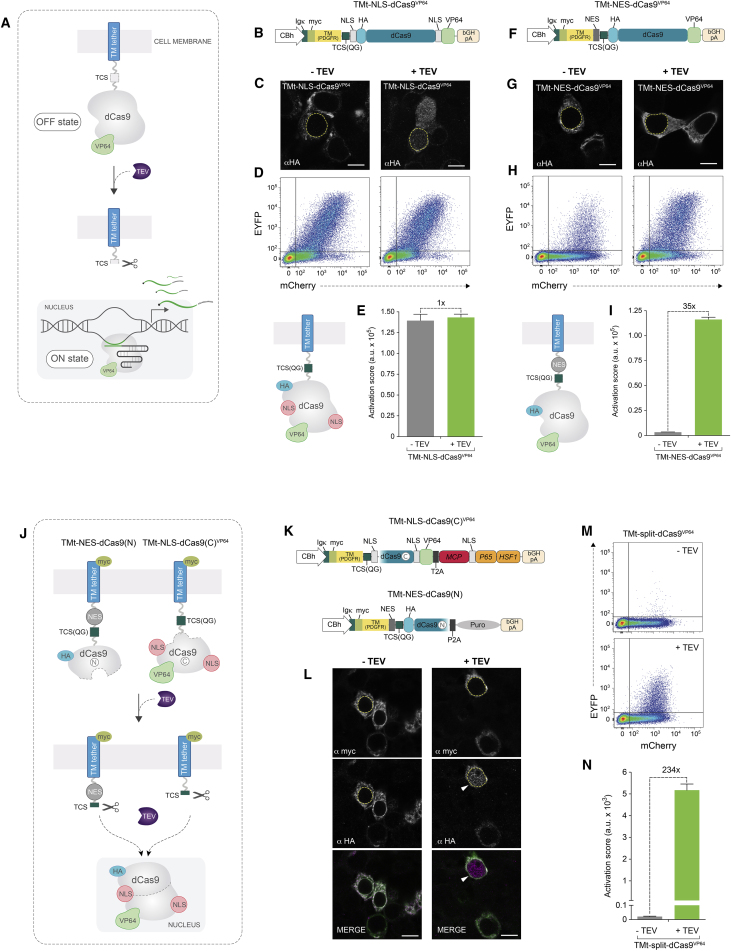
Engineering a Programmable dCas9-VP64-Based Signal Transduction Module (A) Conceptual framework for the implementation of a basic CRISPR-TF membrane tethered module and TEV-based signal release mechanism. (B) Molecular structure of the TMt-NLS-dCas9^VP64^ chimeric construct. (C) Anti-*HA* confocal imaging of HEK293T cells transfected with TMt-NLS-dCas9^VP64^. (D and E) TMt-NLS-dCas9^VP64^ system performance and OFF/ON state transition characteristics measured in the presence or absence of transgenic TEV protease. Representative flow cytometry scatterplots (D) and quantification of EYFP reporter activation score (E) 48 hr after co-transfection of plasmids encoding TMt-NLS-dCas9^VP64^, EYFP reporter, sgEYFP guide RNA, and TEV protease. (F) Schematic representation of TMt-NES-dCas9^VP64^ variant. (G) Immunofluorescence imaging of cells expressing TMt-NES-dCas9^VP64^ stained with anti-*HA* antibody. (H and I) Representative flow cytometry scatterplots of reporter expression (EYFP channel) plotted against sgRNA transfection (mCherry channel) (H) and quantification of corresponding activation scores (see Experimental Procedures and Figure S1) (I). NES membrane tethered dCas9-VP64 variant baseline activation and fold induction following membrane release. (J) Strategy for engineering a split dCas9-VP64 signal transduction module. (K) Structure of split TMt-NES-dCas9(N) and TMt-NLS-dCas9(C)^VP64^ chimeric constructs. TMt-NLS-dCas9(C)^VP64^ plasmid contains the MCP-P65-HSF1 cassette to facilitate future implementation of endogenous gene expression programs. (L) Confocal imaging of the constructs in (K) in the presence and absence of TEV protease, stained by anti-*myc* and anti-*HA* antibodies. (M and N) Analysis of TMt-dCas9(N/C)^VP64^-induced reporter expression by flow cytometry (M) and quantification of corresponding EYFP activation score (N). In all cases the EYFP activation score was calculated from three biological replicates (n = 3 from one experiment, mean ± SD; a.u., arbitrary units). For all confocal images, the dashed yellow line represents nucleus (based on DAPI staining). Scale bar, 10 μm. See also Figures S1 and S2.

We reasoned that a generalizable dCas9-based chimeric receptor architecture should satisfy at least two essential conditions. First, the membrane-tethered dCas9-TF signal-transduction module should display minimal OFF-state baseline activity. Second, the basic receptor scaffold should contain a customizable and broadly applicable intracellular signal-release mechanism that can be engaged upon ligand binding to the extracellular sensing domain. The NIa tobacco etch virus (TEV) protease has been adapted as a highly efficient and versatile tool for studying protein-protein interactions and receptor functions in mammalian cells (Barnea et al., 2008, Kroeze et al., 2015, Wehr et al., 2006). Therefore, we sought to evaluate the potential of using a TEV-released output module for the implementation of dCas9-synRs (Figure 1A). To this end, we first designed a minimal membrane tethered chimeric protein (TMt-NLS-dCas9^VP64^) by grafting a dCas9-VP64 activator to the platelet-derived growth factor (PDGF) receptor transmembrane domain via a short linker containing a canonical TEV cleavage site (TCS) (Figure 1B). In this instance, dCas9-VP64 was flanked by two nuclear localization sequences (NLS) and fused to a HA-epitope tag for subcellular visualization. This construct also encoded an N-terminal cleavable signal peptide (Igκ) required for membrane translocation. Anti-*HA* immunofluorescence analysis of HEK293T cells expressing TMt-NLS-dCas9^VP64^ revealed a cellular distribution characteristic of transmembrane proteins (Figure 1C, −TEV). Accordingly, co-expression of TEV protease resulted in highly efficient release of dCas9-VP64 from the membrane tether and subsequent nuclear localization (Figure 1C, +TEV).

To assess the performance of this minimal design, we employed a well-established fluorescence reporter assay for measuring the activity of dCas9-VP64 transcriptional activators using a single sgRNA (Farzadfard et al., 2013, Ferry et al., 2017, Nissim et al., 2014) (Figure S1). The output of this assay can be converted into an activation score, which integrates both the percentage of activated cells and reporter fluorescence intensity (Figure S1; Experimental Procedures) (Xie et al., 2011). Surprisingly, expression of TMt-NLS-dCas9^VP64^ together with an sgRNA targeting the reporter sites (sgEYFP) revealed robust activation of EYFP expression both in the presence and absence of TEV protease (Figures 1D and 1E). Because TMt-NLS-dCas9^VP64^ is expressed under a strong CBh constitutive promoter, this unexpected leakiness might be a consequence of extensive protein production. To address this possibility, we created a clonal cell line containing a genomically integrated TMt-NLS-dCas9^VP64[Dox]^ transgene under the inducible TRE*tight* promoter (Figure S2A). Analysis of dCas9-VP64-mediated reporter expression relative to promoter induction levels revealed TEV-independent activation even at very low doxycycline concentrations, which was not observed with control sgRNAs (sgSCR) (Figure S2B). Although the fold induction was higher in the presence of TEV protease, this result suggests that the observed TMt-NLS-dCas9^VP64^ background activity is largely independent of the protein levels.

### Design and Optimization of a Split dCas9-Based Signal Transduction Module

Because dCas9-VP64 was engineered as a highly potent transcription factor, the breakdown and reassembly of the nuclear envelope during cell division may allow ectopic activation of target genes. This hypothesis is supported by the observation that the NLS appears to be dispensable for the activity of wild-type (WT) Cas9 in rapidly dividing cells (Oakes et al., 2016). If this was the case, actively transporting the “un-cleaved” dCas9-VP64 out of the nucleus should reduce TEV-independent background activity by limiting the duration of ectopic localization. To test this possibility, we inserted a nuclear export sequence (NES) between the TCS and the transmembrane tether, while also removing the dCas9-VP64 NLS tags (TMt-NES-dCas9^VP64^) (Figure 1F). As expected, immunofluorescence analysis revealed cell membrane distribution of HA-dCas9-VP64 and apparent exclusion from the nucleus both in the presence and absence of TEV protease (Figure 1G). Notably, this new configuration displayed substantially reduced background activity in the absence of TEV and a 35-fold increase in reporter activation score upon TEV expression (Figures 1H and 1I). Confirming the specificity of this effect on TEV-mediated cleavage, a control construct lacking the TCS (TMt-NES^ΔTCS^-dCas9^VP64^) or delivery of a catalytically dead TEV protease (TEV^C151A^) did not show any activity above baseline levels (Figures S2C and S2D). The ability of this construct to drive reporter gene expression even in the absence of an NLS tag is consistent with previous reports (Oakes et al., 2016) and our observation that low levels of dCas9-VP64 are sufficient to promote active transcription.

To further reduce OFF-state background activation and improve system performance, we next engineered the dCas9-VP64 effector complex. Full length Cas9 can be readily split into N-terminal and C-terminal fragments and reassembled to reconstitute an active protein in mammalian cells (Nguyen et al., 2016, Nihongaki et al., 2015, Wright et al., 2015, Zetsche et al., 2015). To evaluate if a split dCas9 architecture could be successfully integrated with our TMt scaffold to enhance its OFF/ON state transition characteristics, we separated the dCas9-VP64 as previously reported (Zetsche et al., 2015) and tethered both fragments to the plasma membrane. Using TCS linkers, we grafted the N-terminal fragment onto TMt-NES and the C-terminal fragment (containing the VP64 effector domain) directly onto the TMt, to generate TMt-NES-dCas9(N) and TMt-NLS-dCas9(C)^VP64^, respectively (Figure 1J). Both constructs carried an N-terminal extracellular myc-tag to visualize membrane localization, while dCas9(N) also encoded an HA-tag for monitoring successful re-assembly events (Figure 1K). Because it was reported that spontaneous dCas9 self-assembly can be a relatively inefficient process (Zetsche et al., 2015), we reintroduced the NLS tags on the C-terminal fragment to promote nuclear translocation of the membrane-released, reconstituted dCas9-VP64 effector complex. To evaluate the performance of this system, we first co-transfected TMt-NES-dCas9(N), TMt-NLS-dCas9(C)^VP64^, and an sgSCR-expressing plasmid in the presence and absence of TEV protease and stained cells with anti-*myc* and anti-*HA* antibodies (Figure 1L). As expected, the extracellular myc-tag was detected on cell membranes reflecting successful translocation of both TMt-NES-dCas9(N) and TMt-NLS-dCas9(C)^VP64^ proteins. The HA-tagged dCas9(N) fragment, however, was localized to the nucleus only in the presence of TEV protease, suggesting spontaneous re-assembly of full-length dCas9-VP64 following membrane release (Figure 1L). Analysis of EYFP reporter expression using the TMt-dCas9(N/C)^VP64^ and the sgEYFP guide RNA revealed minimal OFF-state activity, indicating that this configuration prevents TEV-independent target gene induction, even in rapidly dividing HEK293T cells (Figures 1M and 1N). Importantly, delivery of TEV protease rendered a 234-fold increase in output reporter activation score, demonstrating robust and specific ON-state transition (Figures 1M and 1N). Therefore, this optimized TMt-dCas9(N/C)^VP64^ core scaffold architecture was used for the subsequent design of all dCas9-synR receptors.

### Engineering a Programmable dCas9-synRTK Chimeric Receptor Scaffold

Having optimized a versatile response module and signal-release mechanism, we next sought to create chimeric dCas9-based receptors capable of converting natural extracellular inputs into custom transcriptional outputs. Receptor tyrosine kinases (RTKs) are a well-characterized class of single transmembrane-domain receptors that play essential roles in regulating a variety of cellular functions and have been directly linked to a spectrum of diseases, including cancer, inflammation, and diabetes (Lemmon and Schlessinger, 2010). Most members of this family share a conserved receptor topology, respond to extracellular growth factor signaling, and are activated by ligand-induced dimerization (Lemmon and Schlessinger, 2010). Therefore, we reasoned that chimeric dCas9-synRTKs could enable a vast spectrum of research and therapeutic applications aiming to rewire the cellular information flow.

**Figure 2:**
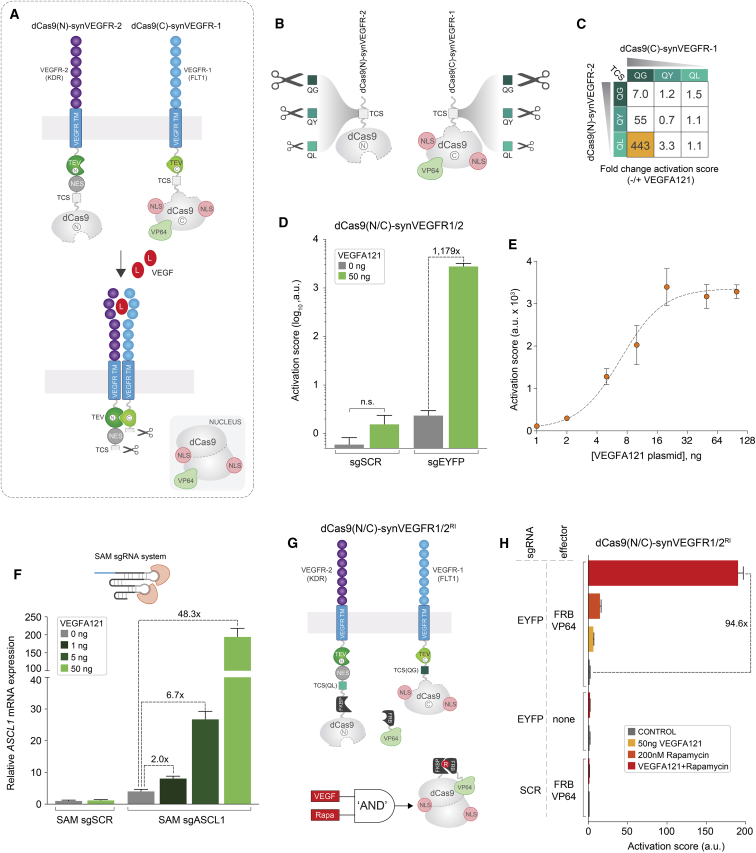
Construction and Optimization of a Prototype Chimeric dCas9-synRTK (A) Design principles underlying the generation of a VEGF-responsive dCas9-synRTK. (B and C) Optimization of chimeric dCas9(N/C)-synVEGFR1/2 performance by fine-tuning coordinated signal release efficiency. Three TCS variants (QG, QY, QL) of decreasing strength were sequentially grafted on both the dCas9(N)-synVEGFR2 and dCas9(C)-synVEGFR1 (B), and the competency of all possible combinations to drive EYFP expression was tested in the presence or absence of VEGFA121 agonist (C) (see Figure S3C). (D) Quantification of EYFP activation score for the top candidate from (C) complemented either with control sgRNA (sgSCR) or targeting sgRNA (sgEYFP), in the presence or absence of VEGFA121 agonist (n = 3 from one experiment, mean ± SD; a.u., arbitrary units; GraphPad Prism unpaired two-sided t test with Welch’s correction, not significant [n.s.] p > 0.05). (E) Dose-response curve for the dCas9(N/C)-synVEGFR1/2 variant in (D) at increasing concentrations of VEGFA121 plasmid. Each data point represents EYFP activation score from 3 biological replicates (mean ± SD, a.u. arbitrary units; curve was fitted using a non-linear variable slope [four parameters] function in GraphPad Prism). (F) Programmed activation of endogenous gene expression by dCas9(N/C)-synVEGFR1/2 in the presence of control SAM sgRNA (SAM sgSCR) or a pool of *ASCL1*-targeting SAM sgRNAs (SAM sgASCL1), and increasing concentrations of VEGFA121 plasmid (n = 3 biological replicates [×3 technical replicates], mean ± SD). (G) Schematic representation of AND gate control for dCas9(N/C)-synVEGFR1/2^RI^ activation. (H) Analysis of EYFP induction by dCas9(N/C)-synVEGFR1/2^RI^ in the absence of any inducer (CONTROL), in the presence of VEGFA121 plasmid alone, rapamycin alone, and combined delivery of both inducers. A control sgRNA (sgRNA SCR) and reactions lacking the FRB-VP64 effector construct were used to establish baseline activation. In all cases the EYFP activation score was calculated from three biological replicates (n = 3 from one experiment, mean ± SD; a.u., arbitrary units; sgSCR, scramble sgRNA control). See also Figure S3.

To engineer a prototype dCas9-synRTK, we selected the vascular endothelial growth factor receptor (VEGFR) family, which contains three closely related members (R1–R3) characterized by extracellular domains composed exclusively of immunoglobulin homology repeats (Olsson et al., 2006). VEGF ligands are soluble, dimeric molecules broadly expressed in various tissues during development and substantially enriched in tumors where they promote angiogenesis (Olsson et al., 2006). VEGFA has been shown to bind with high affinity to VEGFR1 and VEGFR2 homodimers and to VEGFR1/2 heterodimers (Simons et al., 2016). We reasoned that utilizing VEGFR dimerization as a means of controlling TEV activity could yield a self-contained, tightly regulated signal-release mechanism. It was previously reported that the TEV protease could also be segregated in N- and C-terminal inactive fragments and reassembled by complementation into a catalytically active enzyme (Wehr et al., 2006). To this end, we first inserted the N-TEV and C-TEV fragments upstream of NES-dCas9(N) and NLS-dCas9(C)^VP64^, respectively, via a flexible linker (Figure S3A). To identify the most favorable dCas9-synVEGFR architecture, we then grafted the TEV(N)-NES-dCas9(N) and TEV(C)-NLS-dCas9(C)^VP64^ intracellular modules to the native VEGFR1(FLT1) and VEGFR2(KDR) ectodomains via their respective transmembrane helix (Figure S3A; Experimental Procedures). The resulting constructs were delivered to HEK293T cells in a combinatorial fashion and the activity of each homo- and hetero-dimer variant was measured in the presence or absence of transgenically expressed VEGFA121. This analysis revealed that the VEGFR2:TEV(N)-NES-dCas9(N)/VEGFR1:TEV(C)-NLS-dCas9(C)^VP64^ heterodimer displayed the strongest overall output induction (EYFP activation score) and the highest VEGFA121-dependent ON/OFF fold differential (5.5×) (Figure S3B). To simplify nomenclature, these chimeric receptors were subsequently termed dCas9(N)-synVEGFR2 and dCas9(C)-synVEGFR1, respectively. Based on these results, this heterodimer combination was used for all further dCas9(N/C)-synVEGFR optimization and implementation studies (Figure 2A).

Although the dCas9(N/C)-synVEGFR1/2 heterodimer displayed ligand-induced activity, the OFF/ON state transition parameters were inferior to the minimal TMt-dCas9(N/C)^VP64^ design. This may be due to spontaneous dimerization of the extracellular domains, a phenomenon that was previously reported for the native VEGFR2 and other synthetic receptors (Sarabipour et al., 2016, Schwarz et al., 2017). Such proximity-mediated interactions could be particularly problematic for transgenic dCas9-synRs, which are typically expressed under strong promoters. We hypothesized that fine-tuning the kinetics of TEV-mediated signal-release may offset this shortcoming and maximize system performance. For dCas9(N/C)-synVEGFR1/2 this might be accomplished by calibrating the efficiency of the two TCS modules, rendering them competent to license stoichiometric reconstitution of active dCas9-VP64 only upon successful, ligand-mediated receptor activation (i.e., heterodimer stabilization). To test this, we engineered a series of dCas9(N)-synVEGFR2 and dCas9(C)-synVEGFR1 variants with TCS sequences containing point mutations previously reported to decrease TEV binding affinity (ENLYFQ′G > ENLYFQ′Y > ENLYFQ′L) (Barnea et al., 2008, Wang et al., 2017) (Figure 2B). Analysis of all possible variant permutations revealed that coupling NES-dCas9(N) to a low affinity TCS(QL) and NLS-dCas9(C)^VP64^ to a high affinity TCS(QG) substantially improved the specificity of agonist-mediated signal transduction (Figures 2C and S3C). This receptor configuration displayed no significant ligand-independent activity relative to control conditions (sgSCR; scramble sgRNA) and extremely potent output induction upon VEGFA121 expression (up to ∼1,000-fold increase in EYFP activation score) (Figures 2D and S3D). Analysis of target gene induction (EYFP activation score) relative to input agonist levels uncovered a relatively broad VEGFA121 linear response window, indicating that the dCas9-VP64 signal-transduction module can be activated in a sensitive, dose-dependent manner (Figure 2E). This suggests that dCas9-synRTKs could respond to different biological states (i.e., normal versus disease state) by tuning the strength of a custom cellular response relative to the concentration of an extracellular ligand.

Finally, we sought to benchmark the performance of this optimized design against the previously reported VEGF-MESA-dCas9 receptors that contain a full-length dCas9-VP64 intracellular domain (Schwarz et al., 2017). To this end, we transfected each receptor system in HEK293T cells and measured their OFF-state background activation in the absence of agonist. Analysis of EYFP expression revealed that in contrast to dCas9-synVEGFR, in our hands both the V1 and V2 MESA-dCas9 receptors displayed very high background in the un-induced state regardless of the presence or absence of the protease chain (Figure S4). This is consistent with our observation that tethering full-length dCas9-VP64 to the membrane does not provide an adequate framework for engineering an effective signal transduction module, due to high OFF-state leakiness and low signal-to-noise ratio.

### Programmed Endogenous Gene Activation and Inducible Control with dCas9-synRTKs

A defining feature of the dCas9-synR platform is the ability to easily customize the signal-transduction module by simply reprogramming the dCas9-associated sgRNA, which enables actuation of any user-defined endogenous gene expression. Recently, a number of “second-generation” dCas9 activators have been developed to facilitate precise and robust transcriptional control of specific genomic targets with a single sgRNA (Chavez et al., 2016). To investigate if dCas9(N/C)-synVEGFR1/2 could be leveraged to enable induction of a custom endogenous transcriptional response, we programmed it to activate *ASCL1* using previously reported synergistic activation mediator sgRNAs (SAM sgASCL1) (Konermann et al., 2015). Expression of dCas9(N/C)-synVEGFR1/2 heterodimers in the presence of SAM system components and increasing concentrations of VEGFA121 plasmid revealed potent dose-depend induction of *ASCL1* levels, up to 48.3-fold relative to the no-agonist condition (Figure 2F). Notably, this response appeared to be highly specific, as reflected by negligible ligand-independent activation relative to baseline controls (SAM sgSCR).

To incorporate an additional layer of control, we next fused the hetero-dimerization FK506 binding protein 12 (FKBP) domain to dCas9(N), while dissociating the VP64 effector from NLS-dCas9(C) and coupling it to the FKBP rapamycin binding (FRB) domain (Banaszynski et al., 2005, Gao et al., 2016). This resulted in a new receptor variant termed dCas9(N/C)-synVEGFR1/2^RI^. In this case, reconstitution of functional dCas9-VP64 effector fusion is dependent on both an endogenously expressed ligand (VEGFA121) and an extrinsically delivered inducer (rapamycin), thus creating a Boolean “AND” gate logic operator for receptor activation (Figure 2G). In the absence of either inducer, co-delivery of all system components to HEK293T cells showed no detectable reporter expression indicating extremely tight OFF-state control of receptor function (Figure 2H). Similarly, individual delivery of either VEGFA121 or rapamycin failed to generate a notable response. However, concurrent stimulation with both molecules resulted in >90-fold target gene induction demonstrating potent receptor activation and successful implementation of an AND gate function (Figure 2H).

### Design and Implementation of a Ligand-Activated Chimeric dCas9-synGPCR Scaffold

To expand the versatility of dCas9-synRs, we next asked whether the core split-dCas9 architecture could be adapted to integrate other classes of input-sensing modules. G protein-coupled receptors (GPCRs) represent the most extensive superfamily of cell-surface signaling molecules in vertebrates, with functions linked to nearly every physiological process (Dorsam and Gutkind, 2007, Kroeze et al., 2003, Pierce et al., 2002). GPCRs share a conserved seven-transmembrane α-helix topology and can respond to a broad range of extracellular signals including light, small molecules, nucleotides, hormones, lipids, neurotransmitters, and proteins (Pierce et al., 2002). It has been shown for most GPCRs that, in addition to engaging heterotrimeric G protein-mediated canonical signaling, agonist-dependent conformational changes in receptor topology enable phosphorylation by GPCR kinases (GRKs) and subsequent recruitment of β-arrestin2 (Reiter and Lefkowitz, 2006). This basic principle has been exploited to develop a technology termed “transcriptional activation following arrestin translocation” (Tango), which was subsequently adapted for a variety of GPCR-based studies and applications in diverse biological contexts (Barnea et al., 2008, Inagaki et al., 2012, Kroeze et al., 2015, Lee et al., 2017).

**Figure 3:**
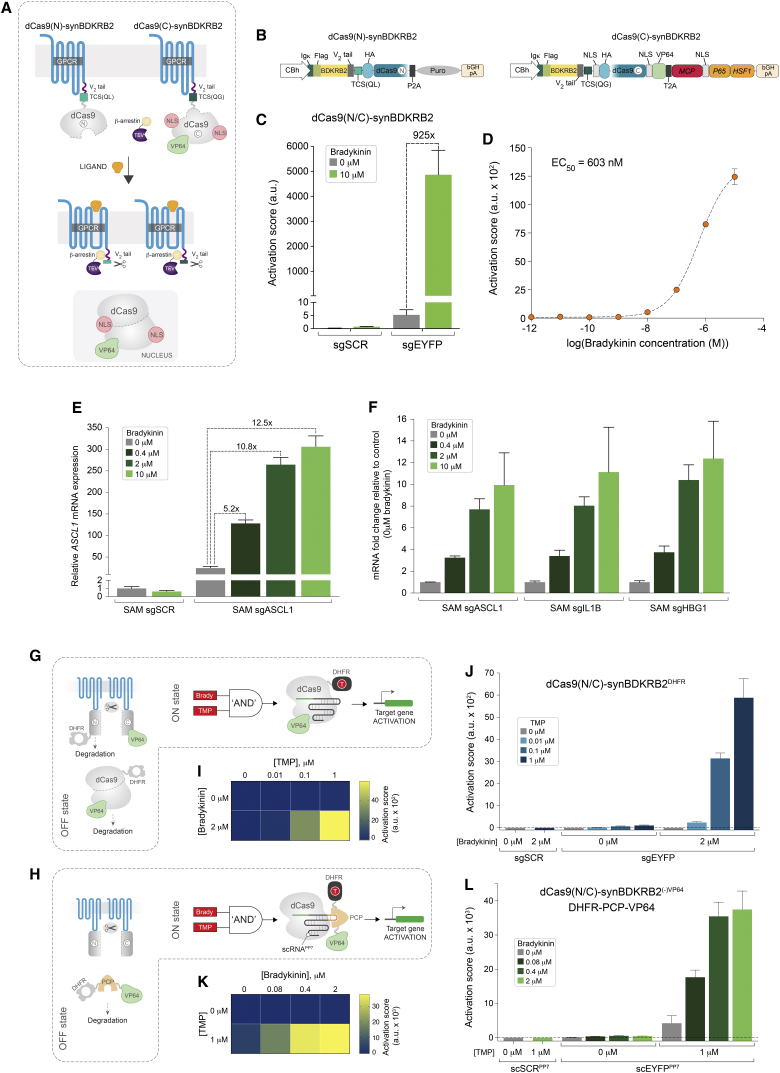
Programmed Control of Gene Expression with Chimeric dCas9-synGPCRs (A) Schematic representation of dCas9-synGPCR design concept. (B) Diagrams of the prototype dCas9(N/C)-synBDKRB2 receptor constructs. (C) Quantification of EYFP activation score following bradykinin-mediated induction of dCas9(N/C)-synBDKRB2 in HTLA cells constitutively expressing β-arrestin2-TEV fusion protein (n = 3 biological replicates from one experiment, mean ± SD, a.u. arbitrary units; sgSCR, scramble sgRNA). (D) Dose-response curve for dCas9(N/C)-synBDKRB2 complemented with sgEYFP guide RNA at increasing concentrations of bradykinin (EC_50_ = half-maximal effective concentration; each data point represents EYFP activation score from 3 biological replicates, mean ± SD, a.u. arbitrary units; curve fitted using a non-linear variable slope [four parameters] function in GraphPad Prism). (E) Induced expression of endogenous *ASCL1* gene by dCas9(N/C)-synBDKRB2 in HTLA cells. Graph shows *ASCL1* mRNA expression levels using a pool of *ASCL1* sgRNAs (SAM sgASCL1) relative to control sgRNA (SAM sgSCR) at increasing concentrations of bradykinin. (F) dCas9(N/C)-synBDKRB2-mediated dose-dependent activation of three target genes (*ASCL1*, *IL1B*, *HBG1*) at increasing agonist concentrations (0.4, 2, 10 μM bradykinin), displayed as fold change relative to no-agonist conditions (0 μM bradykinin). Values in (E) and (F) were calculated from n = 3 biological replicates (×3 technical replicates), mean ± SD. (G and H) Schematic representation of two “AND” gate ON-switch strategies for dCas9(N/C)-synGPCR activation, whereby the DHFR destabilization domain is fused directly to either the dCas9(N) fragment (G) or the PCP-VP64 effector protein (H). (I and J) Heatmap (I) and quantification (J) of reporter activation in the absence or presence of bradykinin and increasing concentrations of TMP, following the strategy described in (G). (K and L) Heatmap (K) and quantification (L) of reporter activation in the absence or presence of TMP and increasing concentrations of bradykinin, following the strategy described in (H). In all cases EYFP activation score was calculated from three biological replicates (n = 3 from one experiment, mean ± SD; a.u., arbitrary units; sgSCR, scramble sgRNA; scSCR^PP7^, scramble PP7-aptamer scaffold guide RNA; scEYFP^PP7^, EYFP-targeting PP7-aptamer scaffold guide RNA). See also Figure S5.

To evaluate the potential of engineering a dCas9-synGPCR, we grafted the NES-dCas9(N):TCS(QL) and NLS-dCas9(C)^VP64^:TCS(QG) modules to the bradykinin GPCR Tango scaffold, to generate dCas9(N)-synBDKRB2 and dCas9(C)-synBDKRB2, respectively (Figures 3A and 3B). A short C-terminal fragment from the *V*_2_ vasopressin receptor tail was inserted before each TCS as previously reported, to enhance β-arrestin2 recruitment (Barnea et al., 2008, Kroeze et al., 2015). Co-transfection of dCas9(N)-synBDKRB2 and dCas9(C)-synBDKRB2 with sgEYFP in a HEK293 cell line constitutively expressing the β-arrestin2-TEV fusion protein (HTLA cells) revealed very tight OFF-state behavior with negligible background receptor activation relative to controls (Figures 3C and S5A). In contrast, addition of bradykinin to the media rendered >900-fold increase in EYFP output activation score, demonstrating potent and specific agonist-mediated signal transduction (Figures 3C and S5A). To establish the dynamic-rage of dCas9(N/C)-synBDKRB2 ligand-mediated induction, we measured output gene expression across increasing concentrations of bradykinin (0.01 nM–10 μM). The ensuing response curve revealed typical dose-dependent activation across a linear range with half-maximal effective agonist concentration (EC_50_) of 603 nM (R^2^ = 0.99, Figure 3D). We also examined the temporal profile of dCas9(N/C)-synBDKRB2 activation by monitoring reporter gene induction at different time points after agonist stimulation. EYFP expression was evident after 12 hr and markedly increased over a period of 24–48 hr, indicating a relatively rapid transduction of the extracellular input signal (bradykinin) into an intracellular output response (Figure S5B).

To determine if this generic architecture can be readily applied to other classes of GPCRs, we appended the split dCas9-VP64 signal transduction module to the AVPR2 and CXCR4 scaffolds, to generate dCas9-synGPCR variants responsive to vasopressin and stromal-derived factor 1 (SDF-1; CXCL12), respectively. The resulting dCas9(N/C)-synAVPR2 and dCas9(N/C)-synCXCR4 chimeric receptors displayed the expected OFF/ON state transition behavior and agonist dose-dependent activation, demonstrating the versatility of this conceptual framework for engineering programmable dCas9-synGPCRs (Figures S5C and S5D).

### Activation of Endogenous Genes and Inducible Control with dCas9-synGPCRs

We next tested the capacity of this prototype dCas9(N/C)-synBDKRB2 to control the expression of an user-defined endogenous gene output by reprogramming its dCas9-VP64 signal-transduction module to target the *ASCL1* genomic locus (SAM-sgASCL1). Analysis of ASCL1 expression as a function of agonist concentration, revealed a robust dose-dependent increase in transcript levels from 5.2-fold to 12.5-fold relative to baseline conditions (Figure 3E). A notable advantage of dCas9-based transcription factors is the ability to drive highly specific and complex gene expression programs by parallel delivery of multiple sgRNAs. Applying this principle in the implementation of dCas9-synRs could enable them to activate custom gene circuit outputs in response to a defined extracellular input. To assess the feasibility of this conceptual framework we have programmed dCas9(N/C)-synBDKRB2 to induce simultaneous activation of three target genes (*ASCL1*, *IL1B*, and *HBG1*) using validated SAM sgRNAs (Konermann et al., 2015) (Figure 3F). Delivery of increasing concentrations of bradykinin revealed potent, dose-dependent induction of all three genes relative to corresponding no-agonist conditions, demonstrating the potential of dCas9-synGPCRs to elicit a complex cellular response (Figure 3F).

To enable exogenous user control over dCas9(N/C)-synGPCR activity, we leveraged a strategy employing small-molecule-regulated protein destabilization domains. The structurally unfolded domain from *Escherichia coli* dihydrofolate reductase (DHFR) has been previously used to promote rapid proteasomal degradation of various fusion proteins including dCas9-TFs (Iwamoto et al., 2010, Maji et al., 2017). In the presence of the small molecule trimethoprim (TMP), DHFR is stabilized in a folded state preventing degradation of the fusion protein (Iwamoto et al., 2010, Maji et al., 2017). To this end, we either fused DHFR to dCas9(N) to generate dCas9(N)-synBDKRB2^DHFR^ (Figure 3G), or used a soluble DHFR-PCP-VP64 effector fusion protein that is recruited to dCas9 via a PP7-aptamer scaffold guide RNA (scRNA^PP7^) (Maji et al., 2017, Zalatan et al., 2015) (Figure 3H). We reasoned that both these systems could facilitate the implementation of “AND” gate logic operators for dCas9(N/C)-synGPCR activation (Figures 3G and 3H). Indeed, analysis of dCas9(N/C)-synBDKRB2^DHFR^ in HTLA cells revealed virtually no activity above baseline in the OFF-state or in the presence of either bradykinin or TMP alone (Figures 3I and 3J). However, concurrent agonist-mediated receptor stimulation and activation with increasing doses of TMP resulted in robust, concentration-dependent reporter gene induction (Figures 3I and 3J). In addition, combining DHFR-PCP-VP64 with a dCas9(N/C)-synBDKRB2^(−)VP64^ variant that lacks VP64 enabled exogenous control of receptor activation over a range of agonist concentrations, although in this case TMP alone could elicit a modest level of induction (Figures 3K and 3L). Together, these experiments demonstrate the integration of two possible “AND” gate logic operators for dCas9(N/C)-synGPCR-mediated cellular reprogramming.

### Implementation of Therapeutically Relevant Cellular Programs with Chimeric dCas9-synRs

**Figure 4:**
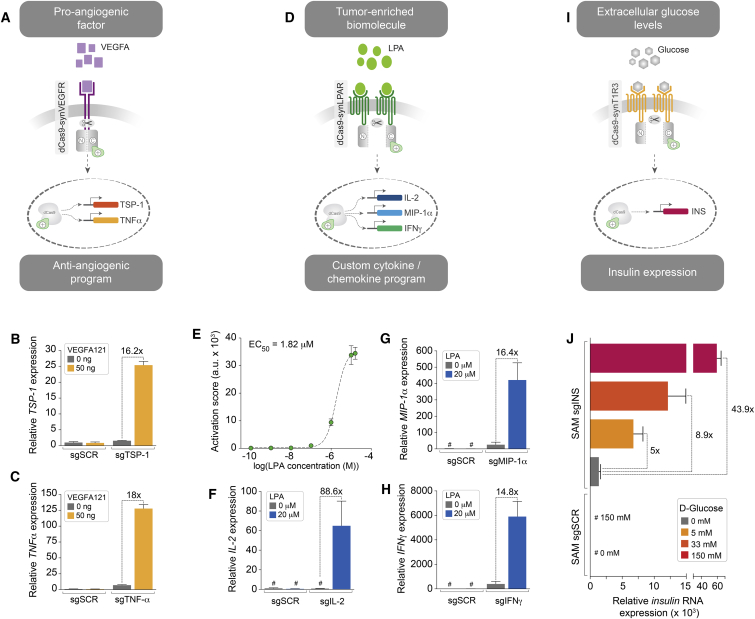
Implementation of Prospective Therapeutic Programs with dCas9-synRs (A) Conversion of a pro-angiogenic signal into a custom anti-angiogenic response by direct reprogramming of the optimized dCas9(N/C)-synVEGFR1/2 receptor with SAM sgRNAs for TSP-1 and TNF-α. (B and C) Real-time qPCR analysis of TSP-1 (B) and TNF-α (C) in HEK293T cells expressing dCas9(N/C)-synVEGFR1/2 receptor and corresponding SAM sgRNAs, in the presence of VEGFA121 plasmid relative to no-agonist controls. (D) LPA-mediated activation of a multifactorial cytokine/chemokine coordinated response in HTLA cells. (E) Analysis of LPA dose-dependent induction of EYFP expression by dCas9(N/C)-synLPAR1 complemented with sgEYFP guide RNA (each data point represents EYFP activation score from 3 biological replicates, mean ± SD, a.u. arbitrary units; curve was fitted using a non-linear variable slope [four parameters] function in GraphPad Prism). (F–H) Quantification of simultaneous dCas9(N/C)-synLPAR1-mediated activation of endogenous IL2 (F), MIP1α (G), and INFγ (H) genes in the presence of exogenously delivered LPA relative to no-agonist conditions. (I) Coupling extracellular glucose levels with programmed insulin expression in HTLA cells. (J) Quantification of insulin transcriptional activation by dCas9(N/C)-synT1R3 following delivery of increasing concentrations of glucose in HTLA cells. Real-time qPCR analysis shows dCas9(N/C)-synT1R3-mediated upregulation of insulin mRNA levels relative to OFF state (no agonist) at physiological glucose concentrations. For all endogenous gene expression analyses n = 3 biological replicates (×3 technical replicates), mean ± SD; sgSCR, control SAM sgRNA; #, undetermined values for the gene of interest were set to a maximum Ct = 40 cycles.

Next, we sought to establish the potential of employing dCas9-synRs to engineer cells that can activate custom therapeutically relevant gene expression programs in response to various disease biomarkers. First, we tested the feasibility of rewiring a pro-angiogenic input signal into a user-defined anti-angiogenic response (Figure 4A). To implement this function, we have re-programmed the dCas9(N/C)-synVEGFR1/2 receptor to drive expression of thrombospondin 1 (TSP-1), a potent inhibitor of angiogenesis, upon VEGFA121-mediated activation (Lawler and Lawler, 2012). Because TSP-1 appears to inhibit VEGFR2 activity without affecting VEGF binding (Kaur et al., 2010), signaling through synthetic dCas9-synVEGFRs should be insensitive to TSP-1, thus enabling implementation of more complex output programs. To test this possibility, we have simultaneously programed induction of a second target gene, the major inflammatory cytokine tumor necrosis factor alpha (TNF-α). Although the impact of TNF-α expression in cancer remains controversial, controlled expression of TNF-α in the tumor microenvironment could be beneficial either by directly targeting the tumor vasculature or by promoting angiostatin biosynthesis (Balkwill, 2009, Burton and Libutti, 2009, Mauceri et al., 2002). Delivery of dCas9(N/C)-synVEGFR1/2 together with previously reported SAM sgRNAs for human TSP-1 and TNF-α revealed potent VEGFA121-mediated induction of both genes compared to endogenous levels (16.2-fold and 18-fold, respectively, relative to no-agonist conditions) (Figures 4B and 4C). As expected, the response of this chimeric receptor to VEGFA121 was highly specific with minimal ligand-independent activation of either gene relative to SAM sgSCR control conditions.

We then used the dCas9-synGPCRs platform to deploy a custom multifactorial cytokine/chemokine coordinated output program (IL2, MIP1α, and INFγ) in response to a soluble extracellular input (lysophosphatidic acid [LPA]) (Figure 4D). LPA is a single fatty acyl chain phospholipid, which has been directly implicated in cancer initiation, progression, and metastasis (Mills and Moolenaar, 2003). LPA is secreted by cancer cells and significantly enriched in the tumor microenvironment, in particular in ovarian and prostate cancers (Mills and Moolenaar, 2003). To engineer an LPA-responsive dCas9-synGPCR, we appended the split dCas9-VP64 signal transduction module to the LPAR1 GPCR Tango scaffold (Kroeze et al., 2015). Demonstrating the portability of the core dCas9-synGPCR architecture, the chimeric dCas9(N/C)-synLPAR1 displayed stringent OFF/ON state transition characteristics with minimal baseline activity and LPA dose-dependent activation (EC_50_ = 1.82 μM, R^2^ = 0.99) (Figure 4E). Programming dCas9(N/C)-synLPAR1 with a combination of IL2, MIP1α, and INFγ SAM sgRNAs resulted in robust LPA-dependent concurrent transcriptional activation of all target genes relative to baseline control levels (Figures 4F–4H). In a prospective therapeutic setting, this program could simultaneously recruit immune cells to the tumor site, promote T cell survival and expansion, and increase the sensitivity of cancer cells to cytotoxic T cells.

Finally, to expand the range of potential dCas9-synR applications, we sought to create a chimeric receptor that could monitor extracellular sugar levels and respond by activating a synthetic circuit resulting in insulin production (Figure 4I). The extracellular Venus flytrap domain of the class C GPCR sweet taste receptor T1R3 has been reported to bind with high affinity glucose and other sugars at physiological concentrations (Nie et al., 2005). To engineer a dCas9(N/C)-synT1R3 receptor, we grafted the split dCas9-VP64 signal transduction module to the T1R3 receptor scaffold via a *V*_2_ tail and corresponding TCS sites as described above. We then programmed dCas9(N/C)-synT1R3 with SAM sgRNAs targeting the endogenous insulin gene and measured output transcriptional activation at various concentrations of D-glucose. This analysis revealed potent glucose-dependent activation of insulin expression in HTLA cells of up to 43-fold compared to baseline no-agonist levels (Figure 4J). Notably, the dCas9(N/C)-synT1R3 receptor rendered a graded dose response in insulin expression (5-fold and 8.9-fold increase) at physiologically relevant D-glucose concentrations (5 mM and 33 mM, respectively) (Nie et al., 2005, Xie et al., 2016) (Figure 4J). Secretion of bioactive insulin, however, will require the implementation of more complex circuits enabling processing of proinsulin into mature insulin and elevation of cytosolic Ca^2+^ concentration (Nishi and Nanjo, 2011, Xie et al., 2016). Nonetheless, these results suggest that receptors such as dCas9(N/C)-synT1R3 may provide a promising biological part for engineering next generations of designer mimetic β-cells for therapeutic applications.

## Discussion

The ability to design artificial signal transduction pathways that can either redirect the information flow or initiate de novo biological programs is profoundly redefining the scope of cellular engineering and its promise in research and therapeutic applications (Lim, 2010, Lim and June, 2017). At the core of this revolutionary dimension of synthetic biology lies the rational assembly and evolution of modular chimeric receptors, which can sense a broad spectrum of natural or synthetic extracellular signals and activate user-defined cellular responses (Lienert et al., 2014, Lim and June, 2017). This basic framework has the potential to enable a wide range of applications including engineering designer therapeutic cells, studying receptor biology, dissecting complex signaling networks, mapping cell-cell interactions, and monitoring environmental perturbations.

Here, we have developed a synthetic receptor paradigm, which enables engineered cells to convert a native signal into direct activation of practically any custom response program. Central to the structural design of dCas9-synRs is a highly customizable and portable signal transduction module consisting of a split dCas9-based transcription factor. To demonstrate the versatility of this conceptual framework, we have created prototype dCas9-synRTKs and dCas9-synGPCRs and tested their capacity to couple diverse soluble input signals (proteins, peptides, lipids, and sugars) with the induction of various user-defined output responses. Although all dCas9-synR variants developed here reproducibly displayed robust agonist-dependent behavior, more work will be needed to expand the versatility of these programmable receptors and realize their broad potential for biomedical research. For example, it should be noted that, in the case of dCas9-synVEGFR, the ligand (VEGFA121) was co-expressed in the same cells, and, consequently, the receptor may have been activated within the secretory pathway while being transported to the plasma membrane. Therefore, future studies will be necessary to characterize and calibrate the response of dCas9-synRTKs to exogenous stimuli or naturally secreted agonists in the context of complex sender/receiver cellular environments. In addition, maintaining a silent OFF-state over sustained periods (e.g., therapeutic settings) and achieving precise spatial-temporal control of ON-state agonist-induced activation may require further optimization of the current dCas9-synR designs with regard to receptor recycling and turnover. Finally, we anticipate that diversifying the dCas9-synR ligand repertoire will occasionally necessitate alterations of the core scaffold, such as including or excluding the *V*_2_ tail in the case of GPCRs (Kroeze et al., 2015) or varying the length and structure of membrane linkers for single transmembrane receptors (e.g., RTKs).

The rationale for engineering dCas9-synRs containing native sensing domains was based on several considerations. First, the extracellular modules of native receptors have been optimized and diversified throughout evolution to bind with high affinity and specificity their cognate ligands. Second, most classes of native receptors possess extremely diverse repertoires of sensing domains but share conserved ligand-induced signal processing mechanisms, thus providing a transferable and flexible modular platform for engineering chimeric receptors. For example, RTKs are activated primarily by agonist-mediated receptor dimerization, while most GPCRs employ a conformational change mechanism to couple ligand binding with recruitment of effector proteins (Lemmon and Schlessinger, 2010, Pierce et al., 2002). Together, these two families of receptors respond to a vast number of soluble ligands, as well other categories of extracellular cues, such as ions, light, and odors. Third, our results suggest that the optimized split-dCas9-TF core module and TEV-based signal release mechanism are applicable to diverse native receptor scaffolds. Consequently, this basic experimental framework could be adapted to enable a variety of additional studies, including deciphering novel receptor functions and dissecting complex cellular interactions during development.

**Figure 5:**
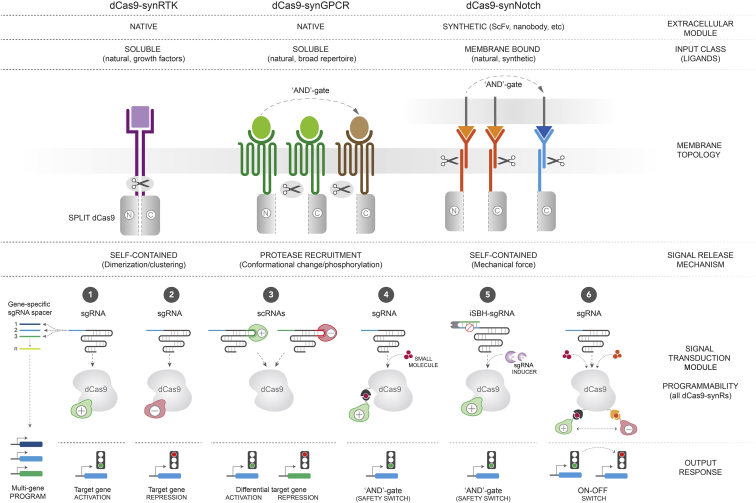
Conceptual Framework for the Evolution of dCas9 Synthetic Receptor Designs The basic split dCas9 signal transduction modular framework offers a highly portable platform for the development of various classes of synthetic receptors containing either native (dCas9-synRTK, dCas9-synGPCR) or artificial (dCas9-synNotch) extracellular input-sensing domains. We show that this architecture is readily adaptable to various signal release mechanisms, including ligand-induced receptor dimerization (RTKs) and conformational change/phosphorylation (GPCRs). In principle, the same design should also be compatible with the force-mediated activation of synNotch receptors, and potentially other types of receptors. The unique versatility of the dCas9 signal transduction module enables dCas9-synRs to couple native or artificial input signals with any custom output response. By multiplexing the number of sgRNAs and using orthogonal effector domains, dCas9-synRs could be programmed to drive sequential or concurrent activation/repression of virtually any endogenous gene. Finally, the recent advent of inducible dCas9 and sgRNA systems facilitates straightforward implementation of various Boolean logic functions, endowing future dCas9-synR variants with a repertoire of tested safety-switch mechanisms.

Notably, however, the universal split dCas9-based signal transduction module developed here is not constrained to native sensing domains, but could be readily integrated in other synthetic receptor designs. For example, the NES-dCas9(N) and NLS-dCas9(C)^VP64^ split domains could be grafted onto the synNotch receptor backbone to replace the Gal4-VP64 or tetR-VP64 transactivators (Morsut et al., 2016) (Figure 5). In this instance, the release of N- and C-terminal dCas9 fragments and subsequent reconstitution of a functional dCas9-VP64 transcriptional activator would be mediated by ligand-induced intramembrane proteolysis. This design could enhance both the input specificity of synNotch receptors by fusing the split dCas9 fragments to heterologous extracellular domains to create dual antigen AND-gates, as well as the versatility of output functions by enabling direct reprogramming of complex combinatorial responses.

An advantage of engineering dCas9-based chimeric receptors is the unique flexibility and functional extensibility of the basic dCas9 scaffold. Owing to recent advances in the CRISPR space, dCas9 has emerged as a master regulator that can be programmed to execute extremely diverse and complex functions by tethering various effector domains to either the dCas9 itself or its associated sgRNA (Jusiak et al., 2016) (Figure 5). In addition, a number of strategies have been devised to spatially and temporally control the regulatory outcome of dCas9-TRs, as well as to enable the implementation of Boolean logic operators in synthetic transcriptional circuits. These include, but are not limited to, post-translational modulation of dCas9 stability and localization, conditional effector tethering and inducible sgRNA systems (Ferry et al., 2017, Gao et al., 2016, Liu et al., 2016, Maji et al., 2017, Nguyen et al., 2016). We show that rendering the assembly of the effector protein (VP64) and dCas9 dependent on a hetero-dimerization system, or using inducible protein destabilization domains, can be employed to integrate AND-gate functions in the core signal transduction module of dCas9-synRs. This system could be further evolved to create even more sophisticated ON/OFF switches by tethering both a transcriptional activator (VPR) and repressor (Krüppel-associated box [KRAB]) to the same dCas9 molecule via previously reported orthogonal hetero-dimerization domains (Gao et al., 2016) (Figure 5). In addition, the activity of dCas9-synRs could also be controlled by recently developed inducible sgRNA platforms, such as signal conductors or iSBH sgRNAs (Ferry et al., 2017, Liu et al., 2016) (Figure 5).

The first generations of dCas9-synRs reported in this study were specifically designed to enable programmed transcriptional activation of target genes by either fusing dCas9 to a VP64 effector domain or coupling it with the more potent SAM system. Alternatively, however, divergent effector proteins (VP64 and KRAB) could be tethered to orthogonal multi-domain scaffold sgRNAs (scRNAs) instead of dCas9, to drive consecutive transcriptional activation and repression of different target genes (Zalatan et al., 2015) (Figure 5). This could enable the implementation of complex logic circuits aimed to enhance the fitness and efficiency of designer therapeutic T cells. For example, T cells engineered to express dCas9-synRs could be programmed with orthogonal scRNAs, to concomitantly drive custom cytokine programs and suppress the transcription of immune co-inhibitory receptors PD-1 and CTLA-4 (Fesnak et al., 2016). Based on all these considerations, we propose that the dCas9-synR platform developed here will provide a valuable template for engineering next generations of programmable chimeric receptors for research and therapeutic applications.

## Experimental Procedures

### Cell Lines and Culture Conditions

HEK293T cells were cultured in DMEM medium (GIBCO) supplemented with 15% (v/v) FBS (GIBCO), 100 U/mL penicillin, and 100 μg/mL streptomycin (GIBCO) (HEK293T full media). HTLA cells were maintained in DMEM supplemented with 10% (v/v) FBS, 100 U/mL penicillin, 100 μg/mL streptomycin, 2 μg/mL puromycin (GIBCO), and 100 μg/mL hygromycin B (GIBCO) (HTLA full media). Plasmids and constructs described in this study are available from Addgene (http://www.addgene.org/Tudor_Fulga/).

### HEK293T and HTLA Transfections

HEK293T or HTLA cells were seeded in 24-well plates (reporter activation assay) or 12-well plates (endogenous gene activation assays and confocal microscopy) and transfected next day at 80%–90% confluency (or ∼70% for confocal imaging). All transfections were performed with Polyethylenimine (PEI Sigma-Aldrich 1 mg/mL). Briefly, plasmids were mixed in either 50 or 100 μL Opti-MEM (GIBCO) for 24-well and 12-well plate transfections, respectively, and PEI was added proportional to the total amount of DNA. A detailed description of the DNA constructs and corresponding amounts used for each transfection reaction is provided in Table S2.

### Flow Cytometry Experiments

Flow cytometry measurements were carried out within 30–60 min from harvest on a BD LSR Fortessa Analyzer (BD Biosciences). The laser configurations and filter sets were maintained across experiments. Forward scatter and side scatter were used to identify the cell population and subsequently live single cells. 100,000 total events were recorded for each condition. Data were analyzed and compensated using the FlowJo package (FLOWJO LLC).

### Real-Time qPCR Analysis

qPCR was carried out using the SsoAdvanced Universal SYBR Green Supermix kit (Cat. #1725272, Bio-Rad) on a CFX384 real-time system (Bio-Rad). Each reaction was run in technical triplicates. In the absence of a relevant PCR product (based on melt curve analysis), values were set to a maximum threshold cycle (Ct) of 40 cycles. Data were analyzed using the ΔΔCt method as previously described (Ferry et al., 2017). A list of all forward and reverse primers used for real-time qPCR analysis is provided in Table S3.

### Confocal Microscopy

For all imaging experiments, cells were fixed in 4% paraformaldehyde (Electron Microscopy Sciences), incubated overnight in 100% EtOH at −20°C, briefly washed, and incubated in blocking buffer (1.5% BSA in 1× TBS). Primary antibodies (polyclonal rabbit HA, Bethyl Laboratories; monoclonal mouse c-*myc*; 9E 10-c, Developmental Studies Hybridoma Bank), secondary antibodies (goat anti-rabbit A488; goat anti-mouse A568, Thermo Fisher Scientific), and DAPI (Invitrogen) were added for 1 hr each at room temperature. Images were acquired on a Zeiss LSM 780 Inverted confocal microscope with an oil immersion objective (Plan-Apochromat 63×/1.4 Oil DIC M27, Zeiss) at non-saturating parameters and processed using the ImageJ package.

## Author Contributions

T.A.B. and T.A.F. conceived the study and designed the experiments. T.A.B. performed the experiments. T.A.B. and T.A.F. analyzed the results. A.A.A. provided essential infrastructure and advice. T.A.B. and T.A.F. wrote the manuscript.

## Supplemental Information

Document S1. Supplemental Experimental Procedures, Figures S1–S5, and Tables S1 and S3

Table S2. Transfection Schemes for Experiments Carried Out in This Study The length of assay refers to the total time from delivery of transfection mixtures to cells until analysis.

Document S2. Article plus Supplemental Information

## References

[bib1] Balkwill F. (2009). Tumour necrosis factor and cancer. Nat. Rev. Cancer.

[bib2] Banaszynski L.A., Liu C.W., Wandless T.J. (2005). Characterization of the FKBP.rapamycin.FRB ternary complex. J. Am. Chem. Soc..

[bib3] Barnea G., Strapps W., Herrada G., Berman Y., Ong J., Kloss B., Axel R., Lee K.J. (2008). The genetic design of signaling cascades to record receptor activation. Proc. Natl. Acad. Sci. USA.

[bib4] Burton E.R., Libutti S.K. (2009). Targeting TNF-alpha for cancer therapy. J. Biol..

[bib5] Chavez A., Tuttle M., Pruitt B.W., Ewen-Campen B., Chari R., Ter-Ovanesyan D., Haque S.J., Cecchi R.J., Kowal E.J.K., Buchthal J. (2016). Comparison of Cas9 activators in multiple species. Nat. Methods.

[bib6] Conklin B.R., Hsiao E.C., Claeysen S., Dumuis A., Srinivasan S., Forsayeth J.R., Guettier J.M., Chang W.C., Pei Y., McCarthy K.D. (2008). Engineering GPCR signaling pathways with RASSLs. Nat. Methods.

[bib7] Dominguez A.A., Lim W.A., Qi L.S. (2016). Beyond editing: repurposing CRISPR-Cas9 for precision genome regulation and interrogation. Nat. Rev. Mol. Cell Biol..

[bib8] Dorsam R.T., Gutkind J.S. (2007). G-protein-coupled receptors and cancer. Nat. Rev. Cancer.

[bib9] Farzadfard F., Perli S.D., Lu T.K. (2013). Tunable and multifunctional eukaryotic transcription factors based on CRISPR/Cas. ACS Synth. Biol..

[bib10] Ferry Q.R., Lyutova R., Fulga T.A. (2017). Rational design of inducible CRISPR guide RNAs for de novo assembly of transcriptional programs. Nat. Commun..

[bib11] Fesnak A.D., June C.H., Levine B.L. (2016). Engineered T cells: the promise and challenges of cancer immunotherapy. Nat. Rev. Cancer.

[bib12] Gao Y., Xiong X., Wong S., Charles E.J., Lim W.A., Qi L.S. (2016). Complex transcriptional modulation with orthogonal and inducible dCas9 regulators. Nat. Methods.

[bib13] Gill S., June C.H. (2015). Going viral: chimeric antigen receptor T-cell therapy for hematological malignancies. Immunol. Rev..

[bib14] Grupp S.A., Kalos M., Barrett D., Aplenc R., Porter D.L., Rheingold S.R., Teachey D.T., Chew A., Hauck B., Wright J.F. (2013). Chimeric antigen receptor-modified T cells for acute lymphoid leukemia. N. Engl. J. Med..

[bib15] Inagaki H.K., Ben-Tabou de-Leon S., Wong A.M., Jagadish S., Ishimoto H., Barnea G., Kitamoto T., Axel R., Anderson D.J. (2012). Visualizing neuromodulation in vivo: TANGO-mapping of dopamine signaling reveals appetite control of sugar sensing. Cell.

[bib16] Iwamoto M., Björklund T., Lundberg C., Kirik D., Wandless T.J. (2010). A general chemical method to regulate protein stability in the mammalian central nervous system. Chem. Biol..

[bib17] Jusiak B., Cleto S., Perez-Piñera P., Lu T.K. (2016). Engineering synthetic gene circuits in living cells with CRISPR technology. Trends Biotechnol..

[bib18] Kaur S., Martin-Manso G., Pendrak M.L., Garfield S.H., Isenberg J.S., Roberts D.D. (2010). Thrombospondin-1 inhibits VEGF receptor-2 signaling by disrupting its association with CD47. J. Biol. Chem..

[bib19] Kershaw M.H., Westwood J.A., Darcy P.K. (2013). Gene-engineered T cells for cancer therapy. Nat. Rev. Cancer.

[bib20] Konermann S., Brigham M.D., Trevino A.E., Joung J., Abudayyeh O.O., Barcena C., Hsu P.D., Habib N., Gootenberg J.S., Nishimasu H. (2015). Genome-scale transcriptional activation by an engineered CRISPR-Cas9 complex. Nature.

[bib21] Kroeze W.K., Sheffler D.J., Roth B.L. (2003). G-protein-coupled receptors at a glance. J. Cell Sci..

[bib22] Kroeze W.K., Sassano M.F., Huang X.P., Lansu K., McCorvy J.D., Giguère P.M., Sciaky N., Roth B.L. (2015). PRESTO-Tango as an open-source resource for interrogation of the druggable human GPCRome. Nat. Struct. Mol. Biol..

[bib23] Lawler P.R., Lawler J. (2012). Molecular basis for the regulation of angiogenesis by thrombospondin-1 and -2. Cold Spring Harb. Perspect. Med..

[bib24] Lee D., Creed M., Jung K., Stefanelli T., Wendler D.J., Oh W.C., Mignocchi N.L., Lüscher C., Kwon H.B. (2017). Temporally precise labeling and control of neuromodulatory circuits in the mammalian brain. Nat. Methods.

[bib25] Lemmon M.A., Schlessinger J. (2010). Cell signaling by receptor tyrosine kinases. Cell.

[bib26] Lienert F., Lohmueller J.J., Garg A., Silver P.A. (2014). Synthetic biology in mammalian cells: next generation research tools and therapeutics. Nat. Rev. Mol. Cell Biol..

[bib27] Lim W.A. (2010). Designing customized cell signalling circuits. Nat. Rev. Mol. Cell Biol..

[bib28] Lim W.A., June C.H. (2017). The Principles of Engineering Immune Cells to Treat Cancer. Cell.

[bib29] Lim W., Mayer B., Pawson T. (2014). Cell Signaling: Principles and Mechanisms.

[bib30] Liu Y., Zhan Y., Chen Z., He A., Li J., Wu H., Liu L., Zhuang C., Lin J., Guo X. (2016). Directing cellular information flow via CRISPR signal conductors. Nat. Methods.

[bib31] Maji B., Moore C.L., Zetsche B., Volz S.E., Zhang F., Shoulders M.D., Choudhary A. (2017). Multidimensional chemical control of CRISPR-Cas9. Nat. Chem. Biol..

[bib32] Mauceri H.J., Seetharam S., Beckett M.A., Lee J.Y., Gupta V.K., Gately S., Stack M.S., Brown C.K., Swedberg K., Kufe D.W., Weichselbaum R.R. (2002). Tumor production of angiostatin is enhanced after exposure to TNF-alpha. Int. J. Cancer.

[bib33] Mills G.B., Moolenaar W.H. (2003). The emerging role of lysophosphatidic acid in cancer. Nat. Rev. Cancer.

[bib34] Morsut L., Roybal K.T., Xiong X., Gordley R.M., Coyle S.M., Thomson M., Lim W.A. (2016). Engineering customized cell sensing and response behaviors using synthetic Notch receptors. Cell.

[bib35] Nguyen D.P., Miyaoka Y., Gilbert L.A., Mayerl S.J., Lee B.H., Weissman J.S., Conklin B.R., Wells J.A. (2016). Ligand-binding domains of nuclear receptors facilitate tight control of split CRISPR activity. Nat. Commun..

[bib36] Nie Y., Vigues S., Hobbs J.R., Conn G.L., Munger S.D. (2005). Distinct contributions of T1R2 and T1R3 taste receptor subunits to the detection of sweet stimuli. Curr. Biol..

[bib37] Nihongaki Y., Kawano F., Nakajima T., Sato M. (2015). Photoactivatable CRISPR-Cas9 for optogenetic genome editing. Nat. Biotechnol..

[bib38] Nishi M., Nanjo K. (2011). Insulin gene mutations and diabetes. J. Diabetes Investig..

[bib39] Nissim L., Perli S.D., Fridkin A., Perez-Pinera P., Lu T.K. (2014). Multiplexed and programmable regulation of gene networks with an integrated RNA and CRISPR/Cas toolkit in human cells. Mol. Cell.

[bib40] Oakes B.L., Nadler D.C., Flamholz A., Fellmann C., Staahl B.T., Doudna J.A., Savage D.F. (2016). Profiling of engineering hotspots identifies an allosteric CRISPR-Cas9 switch. Nat. Biotechnol..

[bib41] Olsson A.K., Dimberg A., Kreuger J., Claesson-Welsh L. (2006). VEGF receptor signalling - in control of vascular function. Nat. Rev. Mol. Cell Biol..

[bib42] Pierce K.L., Premont R.T., Lefkowitz R.J. (2002). Seven-transmembrane receptors. Nat. Rev. Mol. Cell Biol..

[bib43] Reiter E., Lefkowitz R.J. (2006). GRKs and beta-arrestins: roles in receptor silencing, trafficking and signaling. Trends Endocrinol. Metab..

[bib44] Roybal K.T., Rupp L.J., Morsut L., Walker W.J., McNally K.A., Park J.S., Lim W.A. (2016). Precision tumor recognition by T cells with combinatorial antigen-sensing circuits. Cell.

[bib45] Roybal K.T., Williams J.Z., Morsut L., Rupp L.J., Kolinko I., Choe J.H., Walker W.J., McNally K.A., Lim W.A. (2016). Engineering T cells with customized therapeutic response programs using synthetic Notch receptors. Cell.

[bib46] Sarabipour S., Ballmer-Hofer K., Hristova K. (2016). VEGFR-2 conformational switch in response to ligand binding. eLife.

[bib47] Schwarz K.A., Daringer N.M., Dolberg T.B., Leonard J.N. (2017). Rewiring human cellular input-output using modular extracellular sensors. Nat. Chem. Biol..

[bib48] Simons M., Gordon E., Claesson-Welsh L. (2016). Mechanisms and regulation of endothelial VEGF receptor signalling. Nat. Rev. Mol. Cell Biol..

[bib49] Srivastava S., Riddell S.R. (2015). Engineering CAR-T cells: design concepts. Trends Immunol..

[bib50] Turtle C.J., Hanafi L.A., Berger C., Gooley T.A., Cherian S., Hudecek M., Sommermeyer D., Melville K., Pender B., Budiarto T.M. (2016). CD19 CAR-T cells of defined CD4+:CD8+ composition in adult B cell ALL patients. J. Clin. Invest..

[bib51] Wang W., Wildes C.P., Pattarabanjird T., Sanchez M.I., Glober G.F., Matthews G.A., Tye K.M., Ting A.Y. (2017). A light- and calcium-gated transcription factor for imaging and manipulating activated neurons. Nat. Biotechnol..

[bib52] Wehr M.C., Laage R., Bolz U., Fischer T.M., Grünewald S., Scheek S., Bach A., Nave K.A., Rossner M.J. (2006). Monitoring regulated protein-protein interactions using split TEV. Nat. Methods.

[bib53] Wright A.V., Sternberg S.H., Taylor D.W., Staahl B.T., Bardales J.A., Kornfeld J.E., Doudna J.A. (2015). Rational design of a split-Cas9 enzyme complex. Proc. Natl. Acad. Sci. USA.

[bib54] Xie Z., Wroblewska L., Prochazka L., Weiss R., Benenson Y. (2011). Multi-input RNAi-based logic circuit for identification of specific cancer cells. Science.

[bib55] Xie M., Ye H., Wang H., Charpin-El Hamri G., Lormeau C., Saxena P., Stelling J., Fussenegger M. (2016). β-cell-mimetic designer cells provide closed-loop glycemic control. Science.

[bib56] Zalatan J.G., Lee M.E., Almeida R., Gilbert L.A., Whitehead E.H., La Russa M., Tsai J.C., Weissman J.S., Dueber J.E., Qi L.S., Lim W.A. (2015). Engineering complex synthetic transcriptional programs with CRISPR RNA scaffolds. Cell.

[bib57] Zetsche B., Volz S.E., Zhang F. (2015). A split-Cas9 architecture for inducible genome editing and transcription modulation. Nat. Biotechnol..

