# Supplementary material for: Engineering Synthetic Signaling Pathways with Programmable dCas9-Based Chimeric Receptors
Source: Cell Rep. 2017 Sep 12;20(11):2639–53. doi: 10.1016/j.celrep.2017.08.044 (PMC5608971; doi:10.1016/j.celrep.2017.08.044)
Supplement: Document S1. Supplemental Experimental Procedures, Figures S1–S5, and Tables S1 and S3 [file mmc1.pdf]

Cell Reports, Volume *20*

Supplemental Information

Engineering Synthetic Signaling Pathways with Programmable dCas9-Based Chimeric Receptors

Toni A. Baeumler, Ahmed Ashour Ahmed, and Tudor A. Fulga

Figure S1

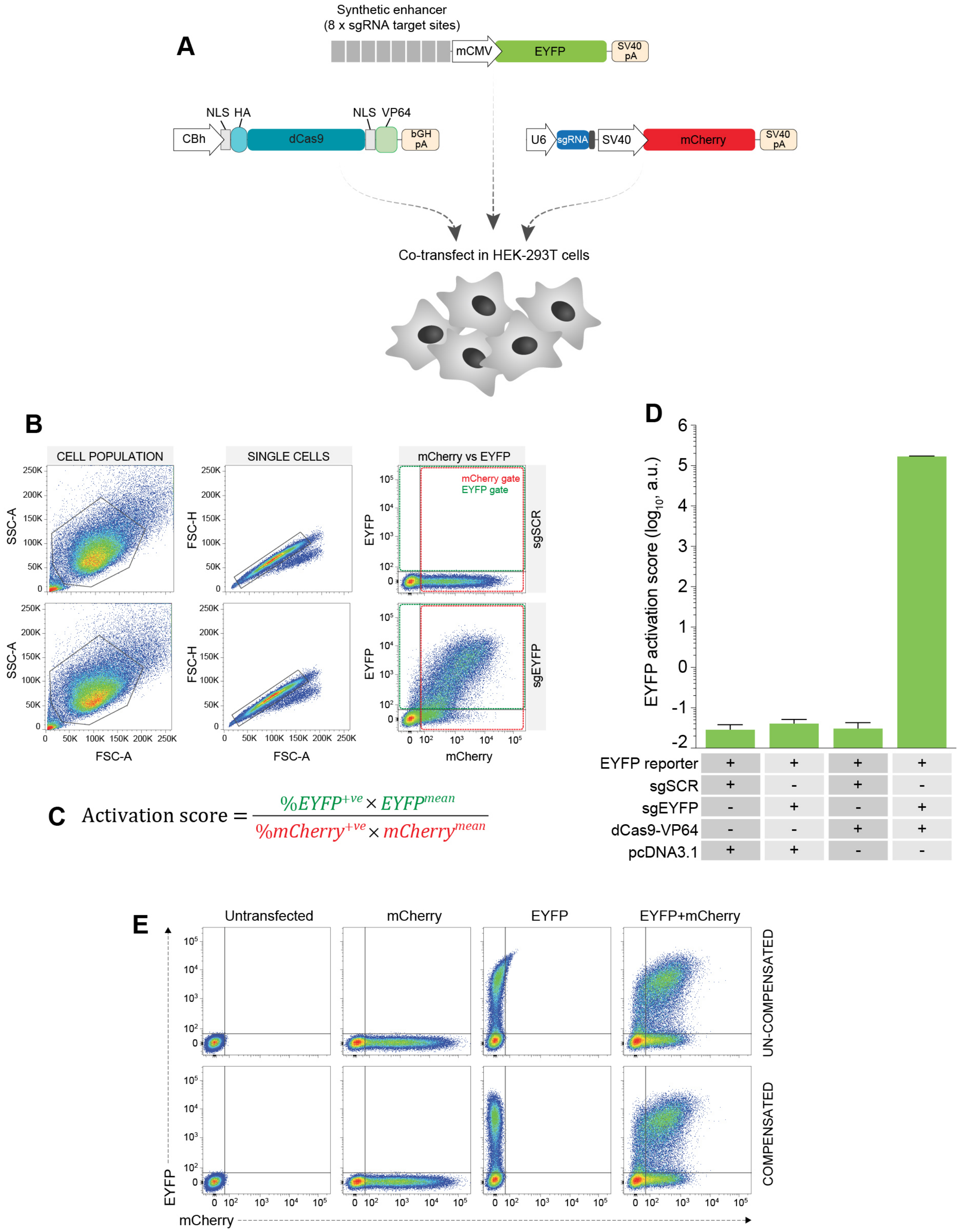

Figure S1. dCas9-VP64 EYFP reporter assay (Related to Experimental Procedures). (A) Schematic representation of the basic EYFP reporter assay. HEK-293T cells were co-transfected with the EYFP reporter plasmid containing a synthetic enhancer (Nissim et al., 2014), dCas9-VP64 expressing plasmid and a plasmid expressing a EYFP-targeting sgRNA and the mCherry transfection control. **(B, C)** Flow cytometry gating strategy and calculation of EYFP activation score. Live cells expressing both EYFP and mCherry were gated as shown in **(B)** and the EYFP activation score was calculated using the formula in **(C)** as previously described (Xie et al., 2011)(see Supplemental Experimental Procedures). Scatter plots show representative raw data for last two conditions in (D). **(D)** Assay specificity. Graph shows EYFP activation score in the presence of all system components (EYFP reporter, dCas9-VP64, sgEYFP guide RNA) compared to control conditions. **(E)** Flow cytometry compensation strategy for dual fluorophore (mCherry/EYFP) analysis. Top row shows un-compensated and bottom row compensated scatter plots.

Figure S2

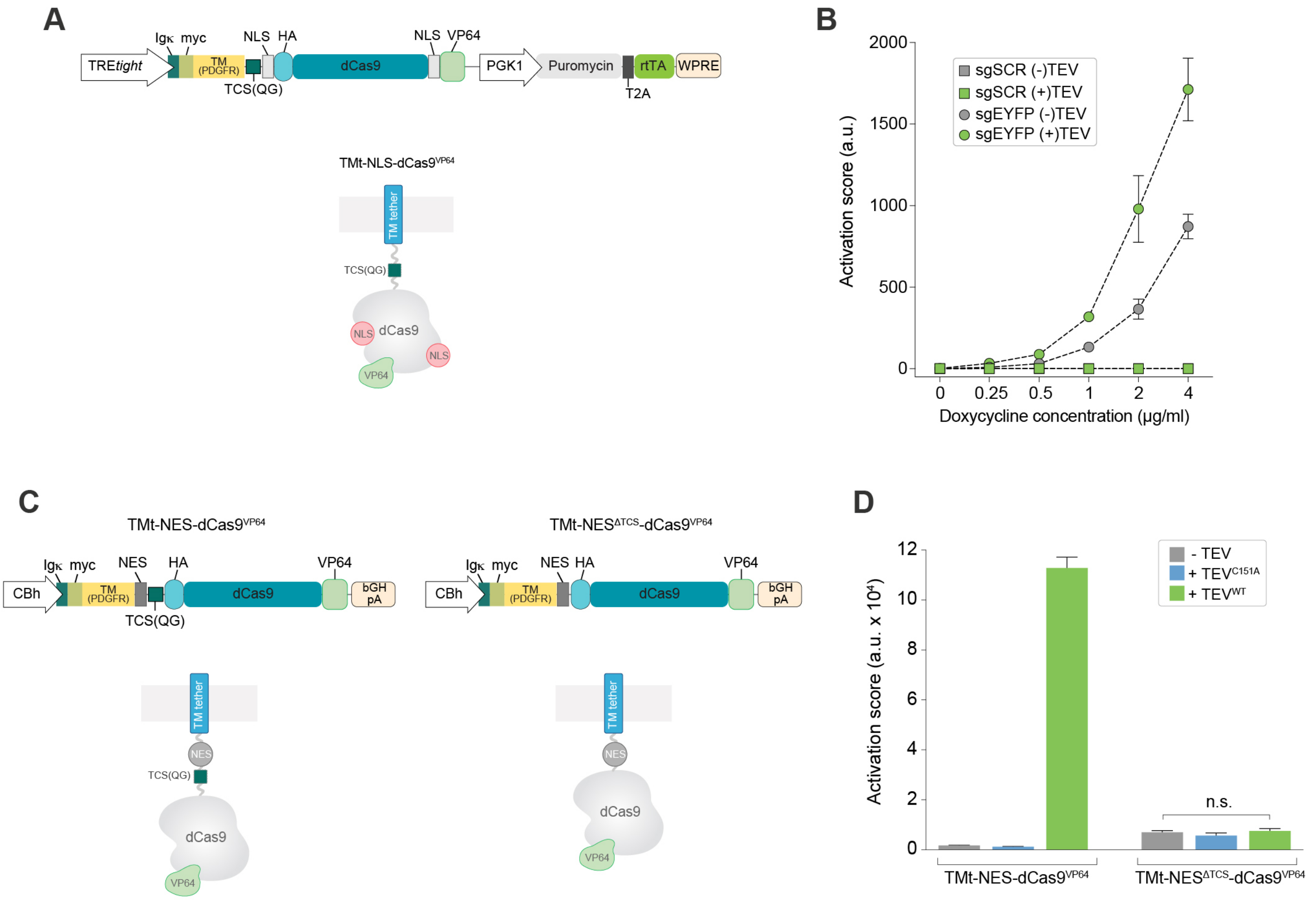

Figure S2. Analysis of TMt-NLS-dCas9^{VP64} and TMt-NES-dCas9^{VP64} performance (Related to Figure 1). (A-B) Impact of TMt-NLS-dCas9^{VP64} expression levels on transcriptional activity. **(A)** Schematic representation of lentiviral vector used for genomic integration. TMt-NLS-dCas9^{VP64} was placed under the doxycycline inducible TREtight promoter to enable controlled expression in HEK-293T cells. This vector constitutively expresses the rtTA transactivator required for TREtight promoter induction. **(B)** Quantification of EYFP reporter activation score at increasing concentration of doxycycline in the presence or absence of co-expressed TEV protease. TMt-NLS-dCas9^{VP64} HEK-293T cells were transfected with plasmids encoding the EYFP reporter, EYFP or control sgRNAs, and TEV protease. 24 hours post-transfection media was supplemented with doxycycline at indicated concentrations for a total of 48 hours. EYFP activation score was calculated from three biological replicates (n = 3 from one experiment, mean +/- s.d.; a.u., arbitrary units; sgSCR = scramble sgRNA control; sgSCR -/+ TEV datapoints overlap). **(C-D)** Specificity of TEV-mediated dCas9-VP64 membrane tether release. **(C)** Schematic diagram of TMt-NES-dCas9^{VP64} and TMt-NES$^{\Delta TCS}$-dCas9^{VP64} constructs. **(D)** Quantification of EYFP activation score in HEK-293T cells transiently transfected with the EYFP reporter, EYFP sgRNA, TMt-NES-dCas9^{VP64} or TMt-NES$^{\Delta TCS}$-dCas9^{VP64} plasmids, in the presence and absence of TEV or mutant TEV^{C151A} protease. EYFP activation score was calculated from three biological replicates (n = 3 from one experiment, mean +/- s.d.; a.u., arbitrary units; GraphPad Prism one way ANOVA test, n.s. $P > 0.05$).

Figure S3

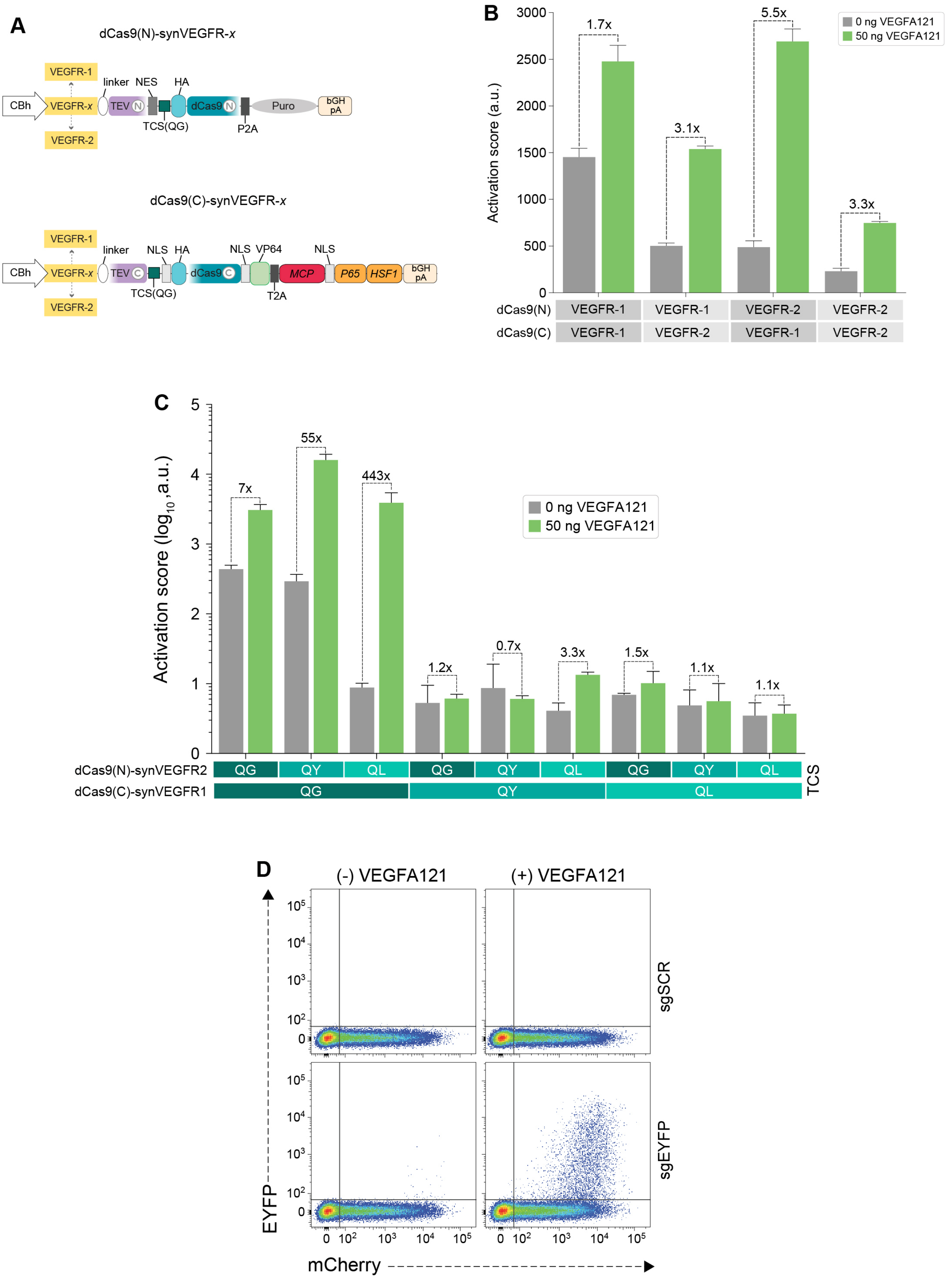

A
dCas9(N)-synVEGFR-x
VEGFR-1
linker NES HA
CBh VEGFR-x TEV N dCas9 N Puro bGH pA
TCS(QG) P2A
VEGFR-2
dCas9(C)-synVEGFR-x
VEGFR-1
linker NLS HA NLS VP64 NLS
CBh VEGFR-x TEV C dCas9 C MCP P65 HSF1 bGH pA
TCS(QG) T2A
VEGFR-2
B
Activation score (a.u.)
0 ng VEGFA121
50 ng VEGFA121
1.7x
3.1x
5.5x
3.3x
dCas9(N) VEGFR-1 VEGFR-1 VEGFR-2 VEGFR-2
dCas9(C) VEGFR-1 VEGFR-2 VEGFR-1 VEGFR-2
C
Activation score (log10,a.u.)
7x
55x
443x
1.2x
0.7x
3.3x
1.5x
1.1x
1.1x
0 ng VEGFA121
50 ng VEGFA121
dCas9(N)-synVEGFR2 QG QY QL QG QY QL QG QY QL
dCas9(C)-synVEGFR1 QG QY QL
TCS
D
(-) VEGFA121
(+) VEGFA121
sgSCR
sgEYFP
EYFP
mCherry

Figure S3. Chimeric dCas9(N/C)-synVEGFR1/2 receptor optimization (Related to Figure 2). (A-B) Identification of optimal dCas9(N/C)-synVEGFR heterodimer configuration. **(A)** Diagrammatic representation of prototype split dCas9(C)-synVEGFR and dCas9(N)-synVEGFR modular constructs highlighting the interchangeable VEGFR-1 (FLT1) and VEGFR-2 (KDR) extracellular domains. The NES-dCas9(N) containing the TCS(QG) motif was fused to the N-terminal TEV fragment and grafted onto the intracellular end of the native VEGFR TM. Similarly, NLS-dCas9(C)^{VP64} containing the TCS(QG) motif was fused to the C-terminal TEV fragment and grafted onto the intracellular end of the native VEGFR TM. **(B)** Quantification of EYFP reporter activation by each dCas9(N/C)-synVEGFR variant programmed with sgEYFP guide RNA, in the presence or absence of co-transfected VEGFA121-expressing plasmid (n = 3 biological replicates from one experiment, mean +/- s.d.; a.u., arbitrary units). **(C)** TCS optimization. Quantification of EYFP reporter activation of all possible dCas9(C)-synVEGFR1 and dCas9(N)-synVEGFR2 TCS variant combinations, in the presence or absence of co-transfected VEGFA121-expressing plasmid (n = 3 biological replicates from one experiment, mean +/- s.d.; a.u., arbitrary units) (related to Figure 2C). **(D)** Representative flow cytometry scatter plots of reporter expression (EYFP channel) plotted against sgRNA transfection (mCherry channel) from cells expressing dCas9(N/C)-synVEGFR1/2 complemented either with control sgRNA (sgSCR) or targeting sgRNA (sgEYFP), in the presence or absence of VEGFA121 agonist (related to Figure 2D).

Figure S4

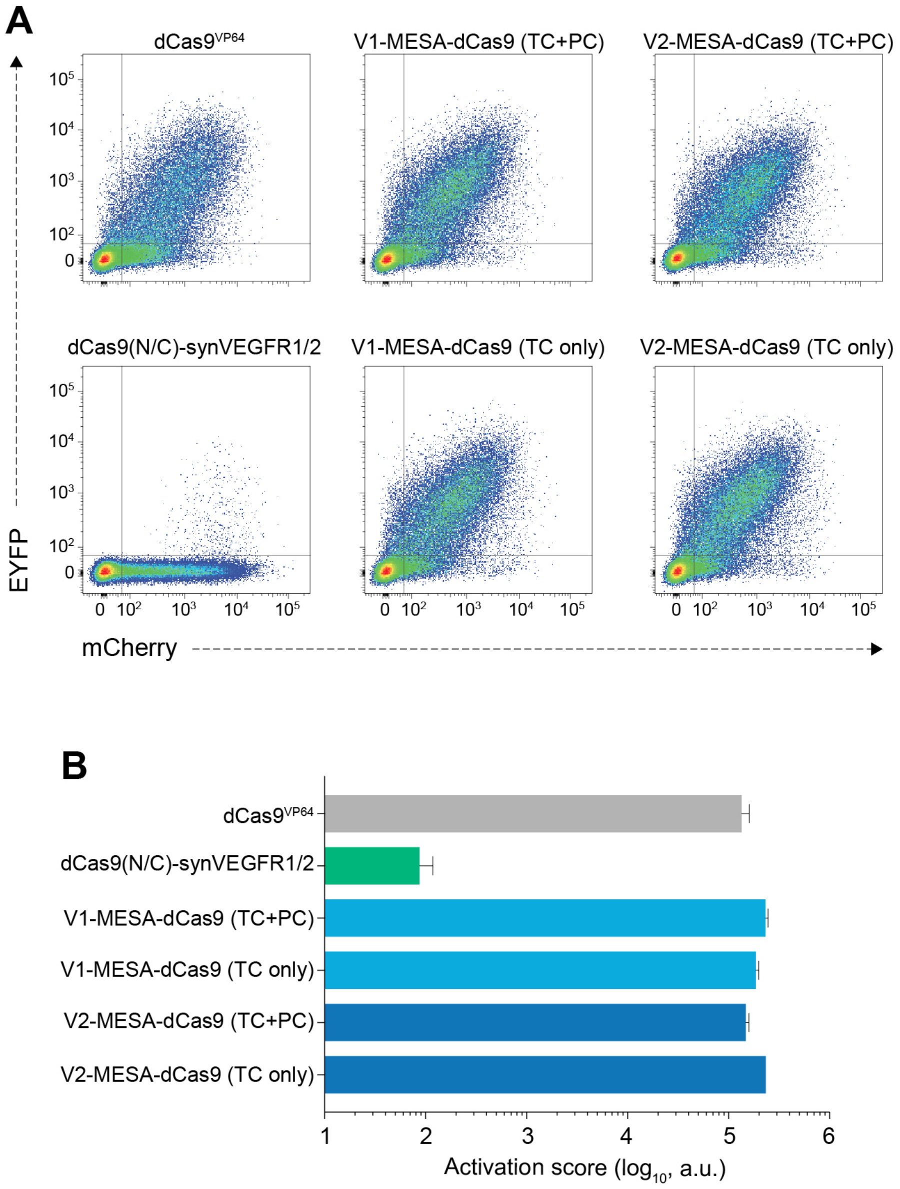

Figure S4. Comparative analysis of dCas9-synVEGFR and MESA-dCas9 OFF-state background activation (Related to Figure 2). (A, B) Representative flow cytometry scatter plots **(A)** and quantification of EYFP reporter activation score **(B)** of cells transfected with full-length dCas9-VP64 (100ng), dCas9(N/C)-synVEGFR1/2 (100ng dCas9(N)-synVEGFR2 + 100ng dCas9(C)-synVEGFR1), V1-MESA-dCas9 and V2-MESA-dCas9 at 10:1 target chain:protease chain ratio (100ng TC + 10ng PC), and V1-MESA-dCas9 and V2-MESA-dCas9 receptors without the protease chain (100ng TC only), in the un-induced state (OFF-state). For a detail description of the MESA receptors as well as the function and ratio of TC and PC transmembrane chains see Schwarz et al., 2017.

Figure S5

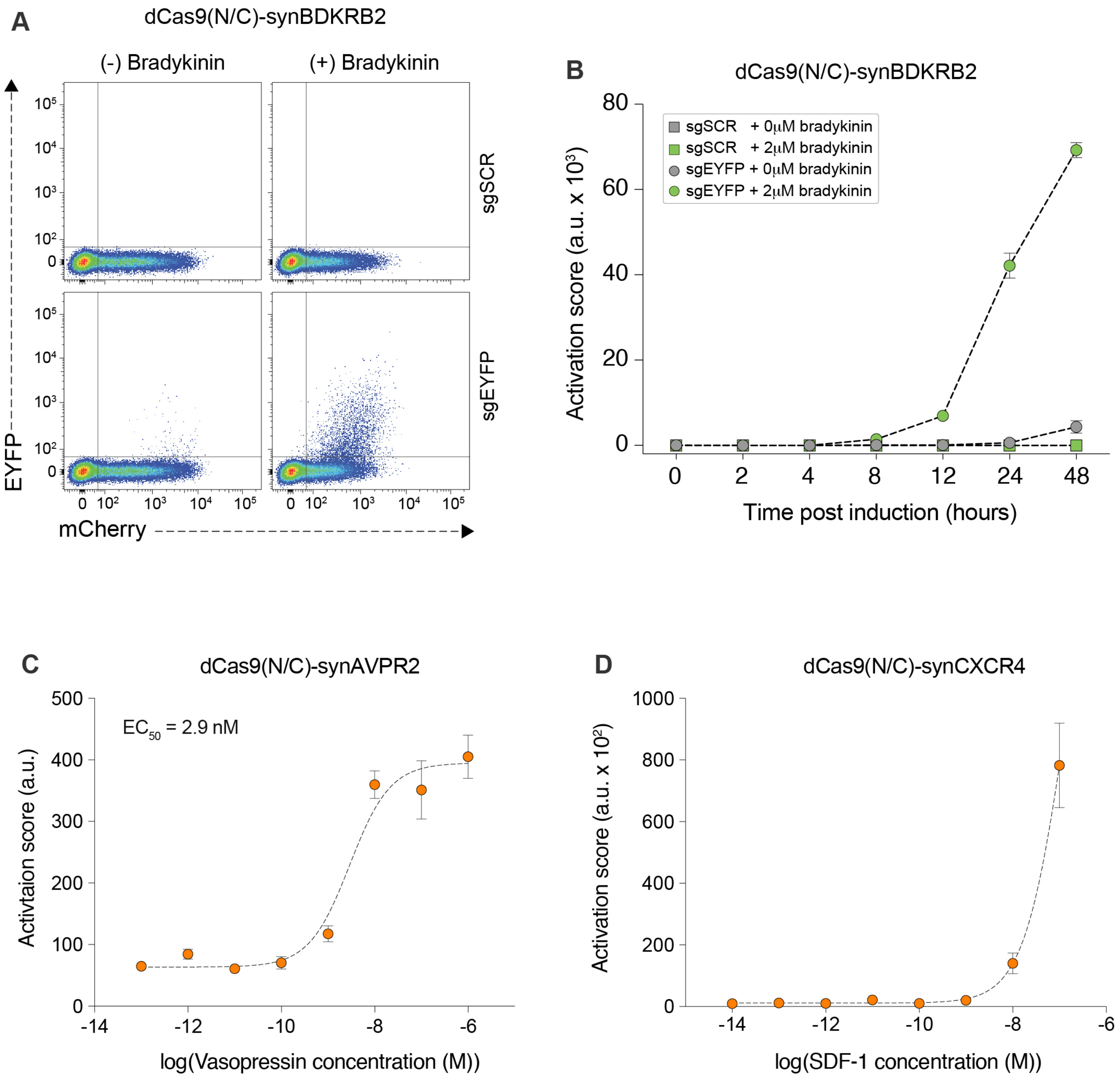

Figure S5. Characterization of different dCas9-synGPCR variants (Related to Figure 3). (A) Representative flow cytometry scatter plots of reporter expression (EYFP channel) plotted against sgRNA transfection (mCherry channel) from cells expressing dCas9(N/C)-synBDKRB2 complemented either with control sgRNA (sgSCR) or targeting sgRNA (sgEYFP), in the presence or absence of Bradykinin (related to Figure 3C). **(B)** Time course analysis of dCas9(N/C)-synBDKRB2 agonist response. HTLA cells were transfected with dCas9(N/C)-synBDKRB2 complemented either with control sgSCR or targeting sgEYFP guide RNAs. After 20 hours, the media was changed to HTLA full media containing no agonist (0 μM bradykinin) or supplemented with 2μM bradykinin. EYFP reporter expression was measured by flow cytometry at 0, 2, 4, 8, 12, 24 and 48 hours post induction. Each time point represents EYFP activation score from three biological replicates, mean +/- s.d., a.u. arbitrary units, sgSCR -/+ bradykinin datapoints overlap. **(C-D)** Vasopressin and SDF-1 responsive dCas9-synGPCRs. Dose-response curves for dCas9(N/C)-synAVPR2 **(C)** and dCas9(N/C)-synCXCR4 **(D)** complemented with sgEYFP guide RNA, at increasing concentrations of vasopressin and SDF-1 (CXCL12), respectively (EC_{50} = half-maximal effective concentration; each data point represents EYFP activation score from 3 biological replicates, mean +/- s.d., a.u. arbitrary units; curve was fitted using a non-linear variable slope (three parameters) function in GraphPad Prism).

Table S1

Target gene	Transcript ID	sgRNA spacer sequence (5'-3')	Reference
ASCL1	NM_004316	GCAGCCGCTCGCTGCAGCAG	Zetsche et al., 2015
		ATGGAGAGTTTGCAAGGAGC	
		GGCTGGGTGTCCCATTGAAA	
		TGTTTATTCAGCCGGGAGTC	
HBG1	NM_000559	GGCTAGGGATGAAGAATAAA	Konermann et al., 2015
		CTTGACCAATAGCCTTGACA	
		AAAATTAGCAGTATCCTCTT	
		GTATCCTCTATGATGGGAGA	
IL1B	NM_000576	TTAGTATATGTGGGACAAAG	
		GAAAATCCAGTATTTTAATG	
		CTCTGGTTCATGGAAGGGCA	
		AGTATTGGTGGAAGCTTCTT	
IL2	NM_000586	ACATCCATTCAGTCAGTCTT	
		ACCCCCAAAGACTGACTGAA	
		GTGGGCTAATGTAACAAAGA	
IFNγ	NM_000619	AACTAAGGTTTTGTGGCATT	
		AAGATGAGATGGTGACAGAT	
		TCTCATCGTCAAAGGACCCA	
MIP-1α (CCL3)	NM_002983	TAGCTCAAAGATGCTATTCT	
		TCAGGGTCCCTGGTGACCAC	
		TTGGATATCCTGAGCCCCTG	
TNFα	NM_000594	GAGAAACCCATGAGCTCATC	
		GGGCCCTGCACCTTCTGTCT	
		TTTCTTCTCCATCGCGGGGG	
TSP-1 (THBS1)	NM_003246	AAAGTGAAGGGGGCGGGGGT	
		GCGGGAGGTGGGGGCCAGTC	
		TAGCTGGAAAGTTGCGCGCC	
INS	NM_001042376	GGGGCTGAGGCTGCAATTTC	Giménez et al., 2016
		CCAGCACCAGGGAAATGGTC	
		CTAATGACCCGCTGGTCCTG	
		AGGTCTGGCCACCGGGCCCC	

Table S1. Sequence of sgRNA spacers used to drive endogenous gene expression in this study. All sgRNAs have been previously reported by Zetsche et al. (*Zetsche, B., Volz, S.E. & Zhang, F. A split-Cas9 architecture for inducible genome editing and transcription modulation. Nat Biotechnol 33, 139-42 (2015)*), Konermann et al. (*Konermann, S. et al. Genome-scale transcriptional activation by an engineered CRISPR-Cas9 complex. Nature 517, 583-8 (2015)*), and Giménez et al. (*Gimenez, C.A. et al. CRISPR-on system for the activation of the endogenous human INS gene. Gene Ther 23, 543-7 (2016)*).

Table S3

qPCR primer	Sequence (5'-3')	Reference
ASCL1 fwd	CCCCAACTACTCCAACGACT	
ASCL1 rev	GGTGAAGTCGAGAAGCTCCT	
GAPDH fwd	AACAGCGACACCCACTCCTC	Ferry, Lyutova, & Fulga, 2017
GAPDH rev	CATACCAGGAAATGAGCTTGACAA	
HBG1 fwd	GTTGTCTACCCATGGACCCA	
HBG1 rev	TCTCCCAAGGAAGTCAGCAC	
IL1B fwd	CGAATCTCCGACCACCACTA	
IL1B rev	AGGGAAAGAAGGTGCTCAGG	
dCas9(C) fwd	AACCTATGCCCACCTGTTCG	
dCas9(C) rev	ATCCAGGATTGTCTTGCCGG	
IL2 fwd	ACCAGGATGCTCACATTTAAGTTTT	Mocellin et al., 2003
IL2 rev	GAGGTTTGAGTTCTTCTTCTAGACAC	
IFNγ fwd	CCAACGCAAAGCAATACATGA	Body-Malapel et al., 2008
IFNγ rev	CCTTTTTCGCTTCCCTGTTTTA	
MIP-1α (CCL3) fwd	GGCTCTCTGCAACCAGTTCT	Lin et al., 2013
MIP-1α (CCL3) rev	TGAAATTCTGTGGAATCTGCC	
INS fwd	ATCAGAAGAGGCCATCAAGCA	Giménez et al., 2016
INS rev	TAGAGAGCTTCCACCAGGTGTGA	
TNFα fwd	CCCAGGGACCTCTCTCTAATCA	Chiu & Yang, 2007
TNFα rev	AGCTGCCCCTCAGCTTGAG	
TSP-1 (THBS1) fwd	ACATGCCACGGCCAACAAA	Ottow et al., 2014
TSP-1 (THBS1) rev	AGTGGCCCAGGTAGTTGCACTT	

Table S3. RT-qPCR primers used in the study.

SUPPLEMENTAL EXPERIMENTAL PROCEDURES

Reagents and kits

Bradykinin acetate salt powder (Cat. #B3259) and Doxycycline hyclate (Cat. #D9891) were purchased from Sigma; 1-oleoyl lysophosphatidic acid (LPA, Cat. #10010093) from Cayman Chemical; D-glucose (Cat. #G/0500/53) from Fisher Scientific; Rapamycin (Cat. #SM83) and Trimethoprim (Cat. #CAY-16473) were purchased from Cambridge Bioscience; vasopressin ([deamino-Cys1, D-Arg8]-Vasopressin acetate salt hydrate) (Cat. #V1005) was purchased from Sigma; recombinant human stromal derived factor 1 (SDF-1α; CXCL12) (Cat. #581202) was purchased from Biolegend. PEI (branched Polyethylenimine, Cat. #408727, Sigma) was diluted in MilliQ water to 1 mg/ml, pH adjusted to 7, sterile filtered and kept in aliquots at -20°C as previously described (Aricescu et al., 2006). All DNA oligonucleotides and PCR primers were obtained from Integrated DNA Technologies (IDT). T4 DNA Ligase (Cat. #M0202), Antarctic phosphatase (Cat. #M0289), T4 Polynucleotide Kinase (Cat. #M0201) and restriction enzymes were purchased from New England Biolabs (NEB) or ThermoFisher Scientific and used according to the manufacturer protocols. PCR reactions were performed using Phusion High-Fidelity PCR Master Mix with GC Buffer (Cat. #M0532, NEB), in a C1000 Thermal Cycler (Bio-Rad). Standard molecular biology techniques and kits were used for all cloning experiments: QIAprep Spin Miniprep Kit (Cat. #27106); QIAfilter Plasmid Midi Kit (Cat. #12243); MinElute PCR Purification Kit (Cat. #28006); QIAquick PCR Purification Kit (Cat. #28106); QIAquick Gel Extraction Kit (Cat. #28706) (Qiagen).

EYFP reporter assay constructs

The following constructs were used for the EYFP reporter assay throughout this study:

Control sgRNA (sgSCR): the sgRNA cassette (*U6 promoter/sgRNA scaffold/U6 terminator*) from pX330 vector (gift from Feng Zhang, Addgene plasmid #42230), f1 origin + SV40 promoter from pcDNA3.1 and mCherry gene (gift from Dr Fabien Pinaud, University of Southern California) were PCR amplified with primers containing MluI(*fwd*) / KpnI(*rev*), KpnI(*fwd*) / NheI(*rev*) and NheI(*fwd*) / EagI(*rev*) respectively, and cloned into the MluI and EagI sites in pcDNA3.1 to generate plasmid *pU6-sgSCR_mCherry*.

EYFP-targeting sgRNA (sgEYFP): the EYFP targeting spacer (5'-GAGTCGCGTGTAGC GAAGCA-3') was synthesised (IDT) and cloned between BbsI sites in the U6-sgSCR_mCherry vector as previously described (Ran et al., 2013) to generate *pU6-sgEYFP_mCherry*.

EYFP reporter: the *P1-EYFP-pA* plasmid containing a synthetic enhancer (8 x target sequences 5'-AGTCGCGTGTAGCGAAGCA-3') recognized by the *sgEYFP* spacer placed upstream of the EYFP reporter gene (gift from Timothy K. Lu, Addgene plasmid #54781), see Figure S1).

NLS-dCas9^{VP64}: The pX330 vector (gift from Feng Zhang, Addgene plasmid #42230) was modified as follows: the *U6 promoter/sgRNA scaffold/U6 terminator* cassette was removed; the FLAG-tag NLS-Cas9 cassette was replaced with dCas9m4-VP64 (gift from George Church, Addgene plasmid #47319) containing a new N-terminal SV40 NLS and HA epitope tag, to generate plasmid

pNLS-HA-dCas9m4-VP64. This vector was only used to establish the EYFP reporter flow cytometry gating strategy (see Figure S1).

dCas9^{VP64}: Cas9m4-VP64 used in Figure S4 was a gift from George Church (Addgene plasmid #47319).

TMt-dCas9, dCas9-synRTK, dCas9-synGPCR and associated constructs

TMt-NLS-dCas9^{VP64}: a transmembrane tether (TMt; modified from the pDisplay Vector (Invitrogen)) containing the Igκ signal sequence, $(GGGS)_2$ linker, myc epitope tag, PDGF receptor transmembrane domain and the XTEN linker (Schellenberger et al., 2009), was synthesized as a gBlock (IDT). This transmembrane tether was then fused to the N-terminus of *dCas9-VP64* via a TEV cleavage site (ENLYFQG) to generate plasmid *pTMt_TCS(Q'G)_NLS-HA-dCas9m4-VP64*.

TMt-NLS-dCas9$^{VP64[Dox]}$: the *TMt-NLS-dCas9^{VP64}* from *pTMt-TCS(Q'G)-NLS-HA-dCas9m4-VP64* was PCR amplified and cloned between XbaI and FseI in pCW-Cas9 (gift from Eric Lander and David Sabatini, Addgene plasmid #50661) to generate pTRE*tight*-TMt_TCS(Q'G)_NLS-dCas9-VP64_PGK1-Puro-T2A-rtTA plasmid.

TMt-NES-dCas9^{VP64}: the NES sequence from pX855 (gift from Feng Zhang, Addgene plasmid #62887) was cloned between the TMt and the TEV cleavage site in *pTMt_TCS(Q'G)_NLS-HA-dCas9m4-VP64*. In addition, the N-terminal NLS of dCas9m4-VP64 was removed while the C-terminal NLS was replaced by a $(GGGS)_2$ linker, to generate plasmid *pTMt_NES_TCS(Q'G)_HA-dCas9m4-VP64*.

TMt-NES$^{\Delta TCS}$-dCas9^{VP64}: the ENLYFQG TEV cleavage site in *pTMt_NES_TCS(Q'G)_HA-dCas9m4-VP64* was replaced by one GGGS linker.

TMt-NES-dCas9(N): the pX855 vector (gift from Feng Zhang, Addgene plasmid #62887) was modified as follows: the *U6 promoter/sgRNA scaffold/U6 terminator* cassette was removed; the dCas9(N) N-terminal NES and the C-terminal FRB+NES were also removed; the TMt-NES-TCS(Q'G)-HA cassette from *pTMt_NES_TCS(Q'G)_HA-dCas9m4-VP64* was fused to the N-terminus of dCas9(N); the puromycin resistance gene and the WPRE stabilising element from pCW-Cas9 (gift from Eric Lander and David Sabatini, Addgene plasmid #50661) were inserted downstream of dCas9(N) via a P2A site to generate plasmid *pTMt_NES_TCS(Q'G)_HA-dCas9(N)_P2A-Puro-WPRE*.

TMt-NLS-dCas9(C)^{VP64}: the pX856 vector (gift from Feng Zhang, Addgene plasmid #62888) was modified as follows: the *U6 promoter/sgRNA scaffold/U6 terminator* cassette was removed; the dCas9(C)^{VP64} N-terminal NLS+FKBP were also removed; the TMt-TCS(Q'G)-NLS-HA cassette from *pTMt_TCS(Q'G)_NLS-HA-dCas9m4-VP64* was fused to the N-terminus of dCas9(C)^{VP64}; the MCP-P65-HSF1 from plasmid MS2-P65-HSF1_GFP (gift from Feng Zhang, Addgene plasmid #61423) was fused to the C-terminus of dCas9(C)^{VP64} via a T2A site to generate plasmid *pTMt_TCS(Q'G)_NLS-HA-dCas9(C)-VP64_T2A_MCP-P65-HSF1*. Note: for confocal imaging

experiments (Figure 1L) the HA tag was removed from this construct.

dCas9(C)-synVEGFR-1: a sequence containing the VEGFR1 (FLT1) leader peptide, extracellular domain and transmembrane domain were PCR amplified from plasmid pDONR223-FLT1 (gift from William Hahn & David Root, Addgene plasmid #23912) and used to replace the TMt in *pTMt_TCS(Q'G)_NLS-HA-dCas9(C)-VP64_T2A_MCP-P65-HSF1.* In addition, the C-terminal TEV fragment was amplified from full length TEV protease as previously described (Wehr et al., 2006) and cloned between the VEGFR1 transmembrane domain and the TEV cleavage site to generate *pVEGFR1_TEV(C)_TCS(Q'G)_NLS-HA-dCas9(C)-VP64_T2A_MCP-P65-HSF1* plasmid.

dCas9(N)-synVEGFR-2: a sequence containing the VEGFR2 (KDR) leader peptide, extracellular domain and transmembrane domain were PCR amplified from plasmid pDONR223-KDR (gift from William Hahn & David Root, Addgene plasmid #23925) and used to replace the TMt in *pTMt_NES_TCS(Q'G)_HA-dCas9(N)_P2A-Puro-WPRE*. The N-terminal TEV fragment was then amplified from full length TEV protease as previously described (Wehr et al., 2006) and fused to the C-terminus of the VEGFR2 transmembrane domain. In addition, a weak TEV cleavage site (ENLYFQL) was inserted instead of the TCS(Q'G) to generate plasmid *pVEGFR2_TEV(N)_NES_TCS(Q'L)_HA-dCas9(N)_P2A_Puro-WPRE*.

For the optimization of *dCas9-synVEGFR* dimer combinations, the VEGFR1 and VEGFR2 PCR products obtained above were interchangeably swapped to generate plasmids *pVEGFR2_TEV(C)_TCS(Q'G)_NLS-HA-dCas9(C)-VP64_T2A_MCP-P65-HSF1* and *pVEGFR1_ TEV(N)_NES_TCS(Q'L)_HA-dCas9(N)_P2A_Puro-WPRE.* For TEV cleavage optimization the TCS in *dCas9(C)-synVEGFR-1* and *dCas9(N)-synVEGFR-2* were iteratively replaced by ENLYFQG, ENLYFQY and ENLYFQL.

dCas9(C)-synVEGFR1^{RI}: the sequences encoding VP64 and T2A-MCP-P65-HSF1 were removed from *pVEGFR1_TEV(C)_TCS(Q'G)_NLS-HA-dCas9(C)-VP64_T2A_MCP-P65-HSF1* to generate *pVEGFR1_TEV(C)_TCS(Q'G)_NLS-HA-dCas9(C).*

dCas9(N)-synVEGFR2^{RI}: the rapamycin inducible hetero-dimerization FK506 binding protein 12 (FKBP) from pX856 vector (gift from Feng Zhang, Addgene plasmid #62888) was fused to the N-terminus of HA-dCas9(N) in *pVEGFR2_TEV(N)_NES_TCS(Q'L)_HA-dCas9(N)_P2A_Puro-WPRE* to generate *pVEGFR2_TEV(N)_NES_TCS(Q'L)_FKBP-HA-dCas9(N)_P2A_Puro-WPRE*.

FRB-VP64: the FKBP rapamycin binding (FRB) from pX855 vector (gift from Feng Zhang, Addgene plasmid #62887) and VP64 were PCR amplified and cloned in between the HindIII and XhoI sites in pcDNA3.1 to generate *pcDNA3.1_FRB-VP64* plasmid.

V1-MESA-dCas9 and *V2-MESA-dCas9:* V1-MESA-45F-M-dCas9, V1-MESA-45F-Tev, V2-MESA-35F-M-dCas9, V2-MESA-35F-Tev were a gift from Joshua Leonard (Addgene plasmid #84504, #84501, #84506, #84503).

dCas9(C)-synBDKRB2: a sequence containing the membrane localisation signal, FLAG tag,

BDKRB2 coding sequence and the V_2 tail were PCR amplified from plasmid BDKRB2-Tango (gift from Bryan Roth, Addgene plasmid #66230) and used to replace the TMt in *pTMt_TCS(Q'G)_NLS-HA-dCas9(C)-VP64_T2A_MCP-P65-HSF1* to generate *pBDKRB2_TCS(Q'G)_NLS-HA-dCas9(C)-VP64_T2A_MCP-P65-HSF1.*

dCas9(N)-synBDKRB2: the TMt, NES and TCS(Q'G) sequences from *pTMt_NES_TCS(Q'G)_HA-dCas9(N)_P2A-Puro-WPRE* were removed and replaced with the membrane localisation signal/FLAG tag/BDKRB2 coding sequence/V_2 tail from plasmid BDKRB2-Tango and the TEV cleavage site ENLYFQL, to generate *pBDKRB2_TCS(Q'L)_HA-dCas9(N)_P2A-Puro-WPRE.*

dCas9(N)-synBDKRB2^{DHFR}: the P2A-Puro-WPRE fragment from *pBDKRB2_TCS(Q'L)_HA-dCas9(N)_P2A-Puro-WPRE* was replaced with DHFR from plasmid *DHFR-PP7-VP64_T2A_GFP* (gift from Amit Choudhary, Addgene plasmid #86167) to generate *pBDKRB2_TCS(Q'L)_HA-dCas9(N)_DHFR.*

dCas9(C)-synBDKRB2$^{(-)VP64}$: the VP64_T2A_MCP-P65-HSF1 cassette from *pBDKRB2_TCS(Q'G)_NLS-HA-dCas9(C)-VP64_T2A_MCP-P65-HSF1* was removed, and DHFR-PCP-VP64 was placed downstream of dCas9(C) sequence separated by a T2A site. The DHFR-PCP-VP64 sequence was obtained from plasmid DHFR-PP7-VP64_T2A_GFP (gift from Amit Choudhary, Addgene plasmid #86167) to generate *pBDKRB2_TCS(Q'G)_NLS-HA-dCas9(C)_T2A_DHFR-PP7-VP64*.

dCas9(C)-synAVPR2: same strategy as *dCas9(C)-synBDKRB2* but instead of BDKRB2, the AVPR2 coding sequence was cloned from plasmid AVPR2-Tango (gift from Bryan Roth (Addgene plasmid #66227)) to generate *pAVPR2_TCS(Q'G)_NLS-HA-dCas9(C)-VP64_T2A_MCP-P65-HSF1*.

dCas9(N)-synAVPR2: same strategy as *dCas9(N)-synBDKRB2* but instead of BDKRB2, the AVPR2 coding sequence was cloned from plasmid AVPR2-Tango (gift from Bryan Roth, Addgene plasmid #66227) to generate *pAVPR2_TCS(Q'L)_HA-dCas9(N)_P2A-Puro-WPRE*.

dCas9(C)-synCXCR4: same strategy as *dCas9(C)-synBDKRB2* but instead of BDKRB2, the CXCR4 coding sequence was cloned from plasmid CXCR4-Tango (gift from Bryan Roth, Addgene plasmid #66262) to generate *pCXCR4_TCS(Q'G)_NLS-HA-dCas9(C)-VP64_T2A_MCP-P65-HSF1*.

dCas9(N)-synCXCR4: same strategy as *dCas9(N)-synBDKRB2* but instead of BDKRB2, the CXCR4 coding sequence was cloned from plasmid CXCR4-Tango (gift from Bryan Roth, Addgene plasmid #66262) to generate *pCXCR4_TCS(Q'L)_HA-dCas9(N)_P2A-Puro-WPRE*.

dCas9(C)-synLPAR1: same strategy as *dCas9(C)-synBDKRB2* but instead of BDKRB2, the LPAR1 coding sequence was cloned from plasmid LPAR1-Tango (gift from Bryan Roth, Addgene plasmid #66418) to generate *pLPAR1_TCS(Q'G)_NLS-HA-dCas9(C)-VP64_T2A_MCP-P65-HSF1*.

dCas9(N)-synLPAR1: same strategy as *dCas9(N)-synBDKRB2* but instead of BDKRB2, the LPAR1 coding sequence was cloned from plasmid LPAR1-Tango (gift from Bryan Roth, Addgene plasmid #66418) to generate *pLPAR1_TCS(Q'L)_HA-dCas9(N)_P2A-Puro-WPRE*.

dCas9(C)-synT1R3: same strategy as *dCas9(C)-synBDKRB2* but instead of BDKRB2, the hT1R3 coding sequence was subcloned from cDNA (gift from Robert Margolskee, Monell Chemical Senses Center, under MTA agreement) to generate *pT1R3_TCS(Q'G)_NLS-HA-dCas9(C)-VP64_T2A_MCP-P65-HSF1*.

dCas9(N)-synT1R3: same strategy as *dCas9(N)-synBDKRB2* but instead of BDKRB2, the hT1R3 coding sequence was subcloned from cDNA (gift from Robert Margolskee, Monell Chemical Senses Center, under MTA agreement) to generate *pT1R3_TCS(Q'L)_HA-dCas9(N)_P2A-Puro-WPRE.*

VEGFA121: the VEGFA121 coding sequence was PCR amplified from pQCXIP-VEGFA121 plasmid (gift from Michael Grusch, Addgene plasmid #73017) and cloned between the HindIII and XhoI sites in pcDNA3.1 to generate *pcDNA3.1_VEGFA121* plasmid.

TEV protease: the TEV protease coding sequence was PCR amplified from plasmid DNA (gift from Dr. Jon Elkins, Nuffield Department of Medicine, University of Oxford) and cloned between BamHI and XhoI in pcDNA3.1 to generate *pcDNA3.1_TEV* plasmid.

SAM sgRNAs: the spacer sequences for all sgRNA targeting endogenous genes were synthesized (IDT) and cloned between BbsI sites in the sgRNA(MS2) cloning backbone (gift from Feng Zhang, Addgene plasmid #61424) as previously described (Ran et al., 2013). All sgRNA spacer sequences used in this study are provided in Table S1. For activation of endogenous genes the corresponding SAM sgRNAs were pooled together and delivered to cells as indicated in Table S2.

scSCR^{PP7}*:* the scaffold RNA containing the PP7 aptamer (gift from Amit Choudhary, Addgene plasmid #86168) was used to replace the sgSCR in *U6-sgSCR_mCherry* vector to create *pU6-scSCR*^{PP7}*_mCherry*.

EYFP-targeting scRNA^{PP7} *(scEYFP*^{PP7}*)*: the EYFP targeting spacer (5'-GAGTCGCGTGTAGC GAAGCA-3') was synthesised (IDT) and cloned between BbsI sites in the *pU6-scSCR*^{PP7}*_mCherry* vector as previously described (Ran et al., 2013) to generate *pU6-scEYFP*^{PP7}*_mCherry*.

Amino acid sequences for representative constructs described here are provided in Supplemental Sequences. DNA constructs were validated by diagnostic restriction digest and/or Sanger sequencing (Source BioScience and Eurofins genomics).

Cell lines and culture conditions details.

HEK-293T cells were purchased from ATCC (ATCC-CRL-11268) and cultured in Dulbecco's modified Eagle's medium (DMEM, Cat. #41966052, Gibco) supplemented with 15% (v/v) FBS (Cat. #10500064, Gibco), 100 U/ml penicillin and 100 µg/ml streptomycin (Cat. #15140122, Gibco)

(HEK-293T full media). HTLA cells (HEK-293 cell line stably expressing a tTA-dependent luciferase reporter and β-arrestin2-TEV fusion protein) were a gift from Bryan Roth. HTLA cells were maintained in DMEM supplemented with 10% (v/v) FBS, 100 U/ml penicillin, 100 µg/ml streptomycin, 2 µg/ml puromycin (Cat. #A1113803, Gibco) and 100 µg/ml hygromycin B (Cat. #10687010, Gibco) (HTLA full media). Both cell lines were cultured at 37°C and 5% CO_2, and passaged every 2 days at 1:6 ratio for 2-3 months before being replaced with a new batch. Cells were infrequently tested for mycoplasma contamination using the Venor®GeM OneStep Mycoplasma Detection Kit (Cat. #11-8025, Minerva Biolabs).

Detailed transfection protocols.

HEK-293T or HTLA cells were seeded in 24-well plates (reporter activation assay) or 12-well plates (endogenous gene activation assays and confocal microscopy) and transfected next day at 80-90% confluency (or approximately 70% for confocal imaging). All transfections were performed with Polyethylenimine (PEI Sigma-Aldrich 1 mg/ml) as previously described(Aricescu et al., 2006). Briefly, plasmids were mixed in either 50 or 100µl Opti-MEM (Cat. #31985047, Gibco) for 24-well and 12-well plate transfections, respectively, and PEI was added proportional to the total amount of DNA as follows: *i)* for experiments in 24-well plates, if the total plasmid concentration was ≤ 600ng, ≤ 800ng, >800ng, the transfection mixtures were supplemented with 1.5µl PEI, 2µl PEI, and 2.5µl PEI, respectively; *ii)* for experiments in 12-well plates, a total plasmid amount of 1µg was used and supplemented with 3µl PEI. Within each experiment, the same amount of DNA was maintained across all conditions by supplementing the transfection mix with pcDNA3.1 where necessary. A detailed description of the DNA constructs and corresponding amounts used for each transfection reaction is provided in Table S2.

Transfection mixtures were vortexed for 10 seconds and incubated at room temperature for 20-30 minutes. Full media was removed from cells and replaced with experiment-specific transfection media prior to adding the DNA:PEI transfection mix as follows. For dCas9(N/C)-synBDKRB2 experiments, HTLA cells were transfected in DMEM + 2% (v/v) FBS supplemented with bradykinin at indicated concentrations. The bradykinin transfection media was replaced after 20 hours with HTLA full media also supplemented with bradykinin, and incubated for an additional 24 hours. For the dCas9(N/C)-synBDKRB2 time course experiment, the HTLA transfection media (without agonist) was replaced after 20 hours with HTLA full media containing no agonist (0 µM bradykinin) or supplemented with 2µM bradykinin. At 0, 2, 4, 8, 12, 24 and 48 hours post bradykinin treatment, HTLA cells were harvested and subjected to flow cytometry analysis (each time point and condition represents three independent transfection experiments). For dCas9(N/C)-synBDKRB2$^{(-)VP64}$ and dCas9(N/C)-synBDKRB2^{DHFR} 'AND' gate experiments, the bradykinin transfection media and bradykinin HTLA full media were also supplemented with trimethoprim (TMP) at indicated concentrations. For dCas9(N/C)-synAVPR2 and dCas9(N/C)-synCXCR4 experiments, the same transfection conditions were used, but the transfection media and HTLA full media were

supplemented with vasopressin or recombinant SDF-1α respectively, at indicated concentrations. For dCas9(N/C)-synLPAR1 experiments, HTLA cells were transfected in DMEM supplemented with 1% (w/v) fatty acid free BSA (Cat. #A8806, Sigma) containing LPA at indicated concentrations. The LPA transfection media was replaced after 20 hours with DMEM 1% (w/v) fatty acid free BSA containing LPA, and incubated for an additional 24 hours. For dCas9(N/C)-synT1R3 experiments, HTLA cells were transfected in DMEM (no glucose, no glutamine, no phenol red, Cat. #A1443001, Gibco) supplemented with 5 mM L-glutamine (Cat. #25030, GIBCO) and 2% (v/v) FBS containing D-glucose at indicated concentrations. The D-glucose transfection media was replaced after 20 hours with DMEM (no glucose, no glutamine, no phenol red), 5 mM L-glutamine and 10% (v/v) FBS containing D-glucose, and incubated for an additional 24 hours.

For all TMt-dCas9 and dCas9-synVEGFR experiments (including the MESA comparison), HEK-293T cells were transfected in DMEM + 2% (v/v) FBS and this media was replaced after 20 or 24 hours with HEK-293T full media for an additional 24 hours. For dCas9(N/C)-synVEGFR1/2^{RI} AND gate experiments, the HEK-293T full media added after transfections also contained rapamycin at indicated concentration. For TMt-NLS-dCas9$^{VP64[Dox]}$ experiments using the stable HEK-293T cell line, transfections were performed in DMEM + 2% (v/v) FBS. Transfection media was changed after 24 hours to HEK-293T full media supplemented with 2 µg/ml puromycin and doxycycline at indicated concentrations for 24 hours, and replaced again with the same media for another 24 hours. For all confocal imaging experiments, HEK-293T cells were directly processed for antibody staining 24 hours after addition of transfection mixtures.

Flow cytometry experiments and data analysis.

For all EYFP reporter experiments, media was removed 44 or 48 hours post-transfection and cells were washed with PBS (1x phosphate buffer saline), trypsinized (0.05% trypsin-EDTA, Cat. #25300062, Gibco), and kept in 1x PBS on ice. Flow cytometry measurements were carried out within 30-60 min from harvest on a BD LSR Fortessa Analyzer (BD Biosciences). The laser configurations and filter sets were maintained across experiments. Forward scatter and side scatter were used to identify the cell population and subsequently live single cells. 100,000 total events were recorded for each condition. Data was analysed and compensated using the FlowJo package (FLOWJO LLC). To calculate an EYFP activation score which integrates both reporter fluorescence intensity and % of activated cells the following formula was used as previously described (Xie et al., 2011).

$$(\%EYFP^{+ve} \times EYFP^{mean})/(\%mCherry^{+ve} \times mCherry^{mean})$$

The numerator ($\%EYFP^{+ve} \times EYFP^{mean}$) provides a weighted mean fluorescence accounting both for the strength of reporter activation ($EYFP^{mean}$) as well as population level activation ($\%EYFP^{+ve}$), which penalizes OFF-state leakage. Since both values are calculated from the parent population (viable single cells) without gating on $mCherry^{+ve}$ cells, the same formula is applied to mCherry for the denominator in order to control for variation in transfection efficiency

($\%mCherry^{+ve}$) and sgRNA levels ($mCherry^{mean}$) between conditions. The fluorescence compensation protocol and the gating strategy used for calculating the EYFP activation score are provided in Figure S1.

It should be noted that due to intrinsic experimental variations (e.g. timing, total amount of plasmids transfected, cell density range, cell passage number) absolute values should only be compared within the same experiment. The variations in fold change activation scores observed for certain constructs is imputable to extremely low (near zero) $\%EYFP^{+ve}$ cells in the OFF-state conditions. For the dose-response curves (bradykinin, SDF-1α, vasopressin and LPA), the lowest concentration plotted represents the no agonist condition.

RT-qPCR analysis

For quantification of endogenous genes expression, transiently transfected cells were harvested 44 hours post-transfection, washed twice in 1x PBS and total RNA was extracted using either RNeasy Mini Kit (Cat. #74106, Qiagen) or EZNA Total RNA Kit I (Cat. #R6834, Omega) following manufacturer's instructions. Complementary DNA (cDNA) was prepared from 1 µg of total RNA using the QuantiTect Reverse Transcription Kit (Cat. #205313, Qiagen). Quantitative PCR (qPCR) was carried out using the SsoAdvanced™ Universal SYBR® Green Supermix kit (Cat. #1725272, Bio-Rad) on a CFX384 real-time system (Bio-Rad). Each reaction was run in technical triplicates. In the absence of a relevant PCR product (based on melt curve analysis), values were set to a maximum Ct of 40 cycles.

Data was analyzed using the ΔΔCt method as previously described (Ferry et al., 2017). ΔCt was calculated using the house keeping gene GAPDH to control for number of cells (GOI transcript levels = 2^(Ct_{GAPDH}-Ct_{GOI})). Before calculating the ΔΔCt, the GOI transcript levels were normalized to dCas9(C) ΔCt for the same condition to account for variations in transfection efficiency (GOI normalized transcript levels = 2^(Ct_{GAPDH}-Ct_{GOI}) / 2^(Ct_{GAPDH}-$Ct_{dCas9(C)}$)). ΔΔCt values for each condition were then calculated and normalized to ΔΔCt in the control conditions (untreated scramble sgRNA) using the formula below (e = experiment (GOI) and c = control (untreated scramble sgRNA, except for Figure 3F where the untreated GOI sgRNAs were used instead)).

$$Fold\ change = 2^{(\mathrm{Ct}^{e}_{GAPDH}-\mathrm{Ct}^{e}_{GOI})-(\mathrm{Ct}^{e}_{GAPDH}-\mathrm{Ct}^{e}_{dCas9(C)})} \Big/ 2^{(\mathrm{Ct}^{c}_{GAPDH}-\mathrm{Ct}^{c}_{GOI})-(\mathrm{Ct}^{c}_{GAPDH}-\mathrm{Ct}^{c}_{dCas9(C)})}$$

A list of all forward and reverse primers used for RT-qPCR analysis is provided in Table S3.

Confocal microscopy

HEK-293T cells were transiently transfected on round coverslips (Cat. #631-1577, VWR), washed twice in 1x PBS, fixed in 4% paraformaldehyde (Cat. #15710, Electron Microscopy Sciences) for 3 min at room temperature and incubated overnight in 100% EtOH at -20°C. EtOH was then removed, cells were briefly washed in washing buffer (1x TBS, 0.2% Triton X-100, 0.04% SDS) and incubated for 1 hour at room temperature in blocking buffer (1.5 % BSA in 1x TBS). Polyclonal

rabbit HA (Cat. #A190-108A, Bethyl Laboratories Inc.) and monoclonal mouse c-myc (9E 10-c, Developmental Studies Hybridoma Bank) primary antibodies were added to cells in blocking buffer and incubated for 1 hour at room temperature. Cells were washed three times in washing buffer and incubated for 1 hour in blocking buffer containing secondary antibodies goat anti-rabbit A488 (Cat. #A-11008, Thermo Fisher Scientific), goat anti-mouse A568 (Cat. #A-11004, Thermo Fisher Scientific) and DAPI (Cat. #D1306, Invitrogen). Coverslips were washed three times in washing buffer and mounted with SlowFade Diamond Antifade Mountant (Cat. #S36972, Life Technologies). Images were acquired on a Zeiss LSM 780 Inverted confocal microscope with an oil immersion objective (Plan-Apochromat 63x/1.4 Oil DIC M27, Zeiss) at non-saturating parameters and processed using the ImageJ package.

Viral production and TMt-NLS-dCas9$^{VP64[Dox]}$ HEK-293T cell line generation

HEK-293T cells were transfected in DMEM + 15% FBS with pCMV-dR8.91 and pMD2.G (gift from Thomas Milne), and TMt-NLS-dCas9$^{VP64[Dox]}$ at a ratio of 1:1:1.5 using Lipofectamine 2000 (Cat. #11668027, Thermo Fisher Scientific). After 24 hours, media was replaced with HEK-293T full media. After another 24 hours, the supernatant containing lentiviral particles was collected, passed through a 0.22 µm filter (Cat. #10268401, Millipore) and added to low passage HEK-293T at low multiplicity of infection (MOI). After 2 days, HEK-293T full media supplemented with 5 µg/ml puromycin was added, transduced cells were passaged three times and then maintained in HEK-293T full media supplemented with 2 µg/ml puromycin. Cells were treated with 1 µg/ml doxycycline, indirectly stained with c-myc primary antibody and goat anti-mouse A568 secondary antibody to identify TMt-NLS-dCas9$^{VP64[Dox]}$ expressing cells, and sorted as single cells into Terasaki plates (Cat. #653180, Greiner Bio-One). One clone displaying the most stringent doxycycline-dependent expression was chosen for subsequent experiments.

SUPPLEMENTAL REFERENCES

Aricescu, A.R., Lu, W., and Jones, E.Y. (2006). A time- and cost-efficient system for high-level protein production in mammalian cells. Acta crystallographica. Section D, Biological crystallography *62*, 1243-1250.

Ferry, Q.R., Lyutova, R., and Fulga, T.A. (2017). Rational design of inducible CRISPR guide RNAs for de novo assembly of transcriptional programs. Nature communications *8*, 14633.

Ran, F.A., Hsu, P.D., Wright, J., Agarwala, V., Scott, D.A., and Zhang, F. (2013). Genome engineering using the CRISPR-Cas9 system. Nature protocols *8*, 2281-2308.

Schellenberger, V., Wang, C.W., Geething, N.C., Spink, B.J., Campbell, A., To, W., Scholle, M.D., Yin, Y., Yao, Y., Bogin, O.*, et al.* (2009). A recombinant polypeptide extends the in vivo half-life of peptides and proteins in a tunable manner. Nature biotechnology *27*, 1186-1190.

Wehr, M.C., Laage, R., Bolz, U., Fischer, T.M., Grunewald, S., Scheek, S., Bach, A., Nave, K.A., and Rossner, M.J. (2006). Monitoring regulated protein-protein interactions using split TEV. Nature methods *3*, 985-993.

Xie, Z., Wroblewska, L., Prochazka, L., Weiss, R., and Benenson, Y. (2011). Multi-input RNAi-based logic circuit for identification of specific cancer cells. Science *333*, 1307-1311.

SUPPLEMENTAL SEQUENCES

mCherry used in *pU6-sgSCR_mCherry and pU6-sgEYFP_mCherry*

MVSKGEEDNMAIIKEFMRFKVHMEGSVNGHEFEIEGEGEGRPYEGTQTAKLKVTKGGPLPFAWDILSPQFMYGSKAYVKH
PADIPDYLKLSFPEGFKWERVMNFEDGGVVTVTQDSSLQDGEFIYKVKLRGTNFPSDGPVMQKKTMGWEASSERMYPEDG
ALKGEIKQRLKLKDGGHYDAEVKTTYKAKKPVQLPGAYNVNIKLDITSHNEDYTIVEQYERAEGRHSTGGMDELYK*

TEV protease used in *pcDNA3.1_TEV*

M**HHHHHHHHH**GESLFKGPRDYNPISSTICHLTNESDGHTTSLYGIGFGPFIITNKHLFRRNNGTLLVQSLHGVFKVKNTT
TLQQHLIDGRDMIIIRMPKDFPPFPQKLKFREPQREERICLVTTNFQTKSMSSMVSDTSCTFPSSDGIFWKHWIQTKDGQ
CGSPLVSTRDGFIVGIHSASNFTNTNNYFTSVPKNFMELLTNQEAQQWVSGWRLNADSVLWGGHKVFMVKPEEPFQPVKE
ATQLMNRRRRP*

NN His9x tag
NN TEV protease sequence

VEGFA121 used in *pcDNA3.1_VEGFA121*

MNFLLSWVHWSLALLLYLHHAKWSQAAPMAEGGGQNHHEVVKFMDVYQRSYCHPIETLVDIFQEYPDEIEYIFKPSCVPL
MRCGGCCNDEGLECVPTEESNITMQIMRIKPHQGQHIGEMSFLQHNKCECRPKKDRARQEKCDKPRR*

FRB-VP64 used in *pcDNA3.1_FRB-VP64*

EMWHEGLEEASRLYFGERNVKGMFEVLEPLHAMMERGPQTLKETSFNQAYGRDLMEAQEWCRKYMKSGNVKDLTQAWDLY
YHVFRRISKQ*GGGSGGGS***GRADALDDFDLDMLGSDALDDFDLDMLGSDALDDFDLDMLGSDALDDFDLDMLIN***

NN FKBP rapamycin binding domain (FRB)
NN (GGGS)$_2$ linker
NN VP64, transcriptional activator domain

TMt-NLS-dCas9^{VP64} used in *pTMt_TCS(Q'G)_NLS-HA-dCas9m4-VP64*

METDTLLLWVLLLWVPGSTGDHS*GGGSGGGS*GRQEQKLISEEDLN***AVGQDTQEVIVVPHSLPFKVVVISAILALVVLTII***
SLIILIMLWQKKPRLQ**SGSETPGTSESATPES**ASHVDHAAA*ENLYFQG*PKKKRKVGGGSTS*YPYDVPDYA*GGSTGMDKKY
SIGLAIGTNSVGWAVITDEYKVPSKKFKVLGNTDRHSIKKNLIGALLFDSGETAEATRLKRTARRRYTRRKNRICYLQEI
FSNEMAKVDDSFFHRLEESFLVEEDKKHERHPIFGNIVDEVAYHEKYPTIYHLRKKLVDSTDKADLRLIYLALAHMIKFR
GHFLIEGDLNPDNSDVDKLFIQLVQTYNQLFEENPINASGVDAKAILSARLSKSRRLENLIAQLPGEKKNGLFGNLIALS
LGLTPNFKSNFDLAEDAKLQLSKDTYDDDLDNLLAQIGDQYADLFLAAKNLSDAILLSDILRVNTEITKAPLSASMIKRY
DEHHQDLTLLKALVRQQLPEKYKEIFFDQSKNGYAGYIDGGASQEEFYKFIKPILEKMDGTEELLVKLNREDLLRKQRTF
DNGSIPHQIHLGELHAILRRQEDFYPFLKDNREKIEKILTFRIPYYVGPLARGNSRFAWMTRKSEETITPWNFEEVVDKG
ASAQSFIERMTNFDKNLPNEKVLPKHSLLYEYFTVYNELTKVKYVTEGMRKPAFLSGEQKKAIVDLLFKTNRKVTVKQLK
EDYFKKIECFDSVEISGVEDRFNASLGTYHDLLKIIKDKDFLDNEENEDILEDIVLTLTLFEDREMIEERLKTYAHLFDD
KVMKQLKRRRYTGWGRLSRKLINGIRDKQSGKTILDFLKSDGFANRNFMQLIHDDSLTFKEDIQKAQVSGQGDSLHEHIA
NLAGSPAIKKGILQTVKVVDELVKVMGRHKPENIVIEMARENQTTQKGQKNSRERMKRIEEGIKELGSQILKEHPVENTQ
LQNEKLYLYYLQNGRDMYVDQELDINRLSDYDVAAIVPQSFLKDDSIDNKVLTRSDKARGKSDNVPSEEVVKKMKNYWRQ
LLNAKLITQRKFDNLTKAERGGLSELDKAGFIKRQLVETRQITKHVAQILDSRMNTKYDENDKLIREVKVITLKSKLVSD
FRKDFQFYKVREINNYHHAHDAYLNAVVGTALIKKYPKLESEFVYGDYKVYDVRKMIAKSEQEIGKATAKYFFYSNIMNF
FKTEITLANGEIRKRPLIETNGETGEIVWDKGRDFATVRKVLSMPQVNIVKKTEVQTGGFSKESILPKRNSDKLIARKKD
WDPKKYGGFDSPTVAYSVLVVAKVEKGKSKKLKSVKELLGITIMERSSFEKNPIDFLEAKGYKEVKKDLIIKLPKYSLFE
LENGRKRMLASAGELQKGNELALPSKYVNFLYLASHYEKLKGSPEDNEQKQLFVEQHKHYLDEIIEQISEFSKRVILADA
NLDKVLSAYNKHRDKPIREQAENIIHLFTLTNLGAPAAFKYFDTTIDRKRYTSTKEVLDATLIHQSITGLYETRIDLSQL
GGDSRADPKKKRKVEAS**GSGRADALDDFDLDMLGSDALDDFDLDMLGSDALDDFDLDMLGSDALDDFDLDMLIN**SR*

NN Igκ, murine Immunoglobin kappa-chain signal peptide
NN (GGGS)$_2$ linker
NN Myc epitope tag
NN TM (PDGFR), transmembrane domain from platelet derived growth factor receptor
NN XTEN linker
NN efficient TCS; tobacco etch virus (TEV) protease cleavage sequence
NN NLS, SV40 nuclear localisation sequence
NN HA, hemagglutinin A epitope
NN dCas9m4, nuclease deficient *Streptococcus pyogenes* Cas9
NN VP64, transcriptional activator domain

TMt-NES-dCas9^{VP64} used in *pTMt_NES_TCS(Q'G)_HA-dCas9m4-VP64*

METDTLLLWVLLLWVPGSTGDHS*GGGSGGGS*GRQEQKLISEEDLN***AVGQDTQEVIVVPHSLPFKVVVISAILALVVLTII***
SLIILIMLWQKKPRLQ**SGSETPGTSESATPES**ASLDLASLILGKLG*ENLYFQG*GGGSTS*YPYDVPDYA*GGSTGMDKKYSI
GLAIGTNSVGWAVITDEYKVPSKKFKVLGNTDRHSIKKNLIGALLFDSGETAEATRLKRTARRRYTRRKNRICYLQEIFS
NEMAKVDDSFFHRLEESFLVEEDKKHERHPIFGNIVDEVAYHEKYPTIYHLRKKLVDSTDKADLRLIYLALAHMIKFRGH
FLIEGDLNPDNSDVDKLFIQLVQTYNQLFEENPINASGVDAKAILSARLSKSRRLENLIAQLPGEKKNGLFGNLIALSLG
LTPNFKSNFDLAEDAKLQLSKDTYDDDLDNLLAQIGDQYADLFLAAKNLSDAILLSDILRVNTEITKAPLSASMIKRYDE
HHQDLTLLKALVRQQLPEKYKEIFFDQSKNGYAGYIDGGASQEEFYKFIKPILEKMDGTEELLVKLNREDLLRKQRTFDN
GSIPHQIHLGELHAILRRQEDFYPFLKDNREKIEKILTFRIPYYVGPLARGNSRFAWMTRKSEETITPWNFEEVVDKGAS
AQSFIERMTNFDKNLPNEKVLPKHSLLYEYFTVYNELTKVKYVTEGMRKPAFLSGEQKKAIVDLLFKTNRKVTVKQLKED
YFKKIECFDSVEISGVEDRFNASLGTYHDLLKIIKDKDFLDNEENEDILEDIVLTLTLFEDREMIEERLKTYAHLFDDKV
MKQLKRRRYTGWGRLSRKLINGIRDKQSGKTILDFLKSDGFANRNFMQLIHDDSLTFKEDIQKAQVSGQGDSLHEHIANL
AGSPAIKKGILQTVKVVDELVKVMGRHKPENIVIEMARENQTTQKGQKNSRERMKRIEEGIKELGSQILKEHPVENTQLQ
NEKLYLYYLQNGRDMYVDQELDINRLSDYDVAAIVPQSFLKDDSIDNKVLTRSDKARGKSDNVPSEEVVKKMKNYWRQLL
NAKLITQRKFDNLTKAERGGLSELDKAGFIKRQLVETRQITKHVAQILDSRMNTKYDENDKLIREVKVITLKSKLVSDFR
KDFQFYKVREINNYHHAHDAYLNAVVGTALIKKYPKLESEFVYGDYKVYDVRKMIAKSEQEIGKATAKYFFYSNIMNFFK
TEITLANGEIRKRPLIETNGETGEIVWDKGRDFATVRKVLSMPQVNIVKKTEVQTGGFSKESILPKRNSDKLIARKKDWD
PKKYGGFDSPTVAYSVLVVAKVEKGKSKKLKSVKELLGITIMERSSFEKNPIDFLEAKGYKEVKKDLIIKLPKYSLFELE
NGRKRMLASAGELQKGNELALPSKYVNFLYLASHYEKLKGSPEDNEQKQLFVEQHKHYLDEIIEQISEFSKRVILADANL
DKVLSAYNKHRDKPIREQAENIIHLFTLTNLGAPAAFKYFDTTIDRKRYTSTKEVLDATLIHQSITGLYETRIDLSQLGG
DGGGSQLGGGS**GSGRADALDDFDLDMLGSDALDDFDLDMLGSDALDDFDLDMLGSDALDDFDLDMLIN**SR*

NN Igκ, murine Immunoglobin kappa-chain signal peptide
NN $(GGGS)_2$ linker
NN Myc epitope tag
NN TM (PDGFR), transmembrane domain from platelet derived growth factor receptor
NN XTEN linker
NN NES, nuclear export signal form human protein tyrosine kinase 2
NN efficient TCS; tobacco etch virus (TEV) protease cleavage sequence
NN HA, hemagglutinin A epitope
NN dCas9m4, nuclease deficient *Streptococcus pyogenes* Cas9
NN VP64, transcriptional activator domain

TMt-NES-dCas9(N) used in *pTMt_NES_TCS(Q'G)_HA-dCas9(N)_P2A-Puro-WPRE*

METDTLLLWVLLLWVPGSTGDHS*GGGSGGGS*GRQEQKLISEEDLN***AVGQDTQEVIVVPHSLPFKVVVISAILALVVLTII***
SLIILIMLWQKKPRLQ**SGSETPGTSESATPES**ASLDLASLILGKLG*ENLYFQG*GGGSTS*YPYDVPDYA*GGSGSDKKYSIG
LAIGTNSVGWAVITDEYKVPSKKFKVLGNTDRHSIKKNLIGALLFDSGETAEATRLKRTARRRYTRRKNRICYLQEIFSN
EMAKVDDSFFHRLEESFLVEEDKKHERHPIFGNIVDEVAYHEKYPTIYHLRKKLVDSTDKADLRLIYLALAHMIKFRGHF
LIEGDLNPDNSDVDKLFIQLVQTYNQLFEENPINASGVDAKAILSARLSKSRRLENLIAQLPGEKKNGLFGNLIALSLGL
TPNFKSNFDLAEDAKLQLSKDTYDDDLDNLLAQIGDQYADLFLAAKNLSDAILLSDILRVNTEITKAPLSASMIKRYDEH
HQDLTLLKALVRQQLPEKYKEIFFDQSKNGYAGYIDGGASQEEFYKFIKPILEKMDGTEELLVKLNREDLLRKQRTFDNG
SIPHQIHLGELHAILRRQEDFYPFLKDNREKIEKILTFRIPYYVGPLARGNSRFAWMTRKSEETITPWNFEEVVDKGASA
QSFIERMTNFDKNLPNEKVLPKHSLLYEYFTVYNELTKVKYVTEGMRGGGGSGTGS**GATNFSLLKQAGDVEENPGP**EF*MT*
EYKPTVRLATRDDVPRAVRTLAAAFADYPATRHTVDPDRHIERVTELQELFLTRVGLDIGKVWVADDGAAVAVWTTPESV
EAGAVFAEIGPRMAELSGSRLAAQQQMEGLLAPHRPKEPAWFLATVGVSPDHQGKGLGSAVVLPGVEAAERAGVPAFLET
*SAPRNLPFYERLGFTVTADVEVPEGPRTWCMTRKG**

NN Igκ, murine Immunoglobin kappa-chain signal peptide
NN $(GGGS)_2$ linker
NN Myc epitope tag
NN TM (PDGFR), transmembrane domain from platelet derived growth factor receptor
NN XTEN linker
NN NES, nuclear export signal form human protein tyrosine kinase 2
NN efficient TCS; tobacco etch virus (TEV) protease cleavage sequence
NN HA, hemagglutinin A epitope
NN dCas9(N), N-terminal moiety of human codon–optimized *Streptococcus pyogenes* Cas9
NN P2A, 2A self-cleaving peptide from porcine teschovirus-1
NN Puromycin resistance protein

TMt-NLS-dCas9(C)^{VP64} used in *pTMt_TCS(Q'G)_NLS-HA-dCas9(C)-VP64_T2A_MCP-P65-HSF1*

METDTLLLWVLLLWVPGSTGDHSGGGSGGGSGRQEQKLISEEDLNAVGQDTQEVIVVPHSLPFKVVVISAILALVVLTII
SLIILIMLWQKKPRLQSGSETPGTSESATPESASHVDHAAAENLYFQGPKKKRKVGGGSTSYPYDVPDYAGGSGSGGGSK
PAFLSGEQKKAIVDLLFKTNRKVTVKQLKEDYFKKIECFDSVEISGVEDRFNASLGTYHDLLKIIKDKDFLDNEENEDIL
EDIVLTLTLFEDREMIEERLKTYAHLFDDKVMKQLKRRRYTGWGRLSRKLINGIRDKQSGKTILDFLKSDGFANRNFMQL
IHDDSLTFKEDIQKAQVSGQGDSLHEHIANLAGSPAIKKGILQTVKVVDELVKVMGRHKPENIVIEMARENQTTQKGQKN
SRERMKRIEEGIKELGSQILKEHPVENTQLQNEKLYLYYLQNGRDMYVDQELDINRLSDYDVDHIVPQSFLKDDSIDNKV
LTRSDKARGKSDNVPSEEVVKKMKNYWRQLLNAKLITQRKFDNLTKAERGGLSELDKAGFIKRQLVETRQITKHVAQILD
SRMNTKYDENDKLIREVKVITLKSKLVSDFRKDFQFYKVREINNYHHAHDAYLNAVVGTALIKKYPKLESEFVYGDYKVY
DVRKMIAKSEQEIGKATAKYFFYSNIMNFFKTEITLANGEIRKRPLIETNGETGEIVWDKGRDFATVRKVLSMPQVNIVK
KTEVQTGGFSKESILPKRNSDKLIARKKDWDPKKYGGFDSPTVAYSVLVVAKVEKGKSKKLKSVKELLGITIMERSSFEK
NPIDFLEAKGYKEVKKDLIIKLPKYSLFELENGRKRMLASAGELQKGNELALPSKYVNFLYLASHYEKLKGSPEDNEQKQ
LFVEQHKHYLDEIIEQISEFSKRVILADANLDKVLSAYNKHRDKPIREQAENIIHLFTLTNLGAPAAFKYFDTTIDRKRY
TSTKEVLDATLIHQSITGLYETRIDLSQLGGDSPKKKRKVEASGRADALDDFDLDMLGSDALDDFDLDMLGSDALDDFDL
DMLGSDALDDFDLDMLINGTASGSGEGRGSLLTCGDVEENPGPVSKLMASNFTQFVLVDNGGTGDVTVAPSNFANGVAEW
ISSNSRSQAYKVTCSVRQSSAQKRKYTIKVEVPKVATQTVGGVELPVAAWRSYLNMELTIPIFATNSDCELIVKAMQGLL
KDGNPIPSAIAANSGIYSAGGGGSGGGGSGGGGSGPKKKRKVAAAGSPSGQISNQALALAPSSAPVLAQTMVPSSAMVPL
AQPPAPAPVLTPGPPQSLSAPVPKSTQAGEGTLSEALLHLQFDADEDLGALLGNSTDPGVFTDLASVDNSEFQQLLNQGV
SMSHSTAEPMLMEYPEAITRLVTGSQRPPDPAPTPLGTSGLPNGLSGDEDFSSIADMDFSALLSQISSSGQGGGGSGFSV
DTSALLDLFSPSVTVPDMSLPDLDSSLASIQELLSPQEPPRPPEAENSSPDSGKQLVHYTAQPLFLLDPGSVDTGSNDLP
VLFELGEGSYFSEGDGFAEDPTISLLTGSEPPKAKDPTVS*

NN Igκ, murine Immunoglobin kappa-chain signal peptide
NN $(GGGS)_2$ linker
NN Myc epitope tag
NN TM (PDGFR), transmembrane domain from platelet derived growth factor receptor
NN XTEN linker
NN efficient TCS; tobacco etch virus (TEV) protease cleavage sequence
NN NLS, SV40 nuclear localisation sequence
NN HA, hemagglutinin A epitope
NN dCas9(C), C-terminal moiety of human codon–optimized *Streptococcus pyogenes* Cas9
NN VP64, transcriptional activator domain
NN T2A, 2A self-cleaving peptide from thosea asigna virus
NN MCP (MS2 protein), MS2 bacteriophage coat protein
NN P65, activation domain from human NF-kB trans-activating subunit p65
NN HSF1, activation domains from human heat-shock factor 1

dCas9(C)-synVEGFR-1 used in *pVEGFR1_TEV(C)_TCS(Q'G)_NLS-HA-dCas9(C)-VP64_T2A_MCP-P65-HSF1*

MVSYWDTGVLLCALLSCLLLTGSSSGSKLKDPELSLKGTQHIMQAGQTLHLQCRGEAAHKWSLPEMVSKESERLSITKSA
CGRNGKQFCSTLTLNTAQANHTGFYSCKYLAVPTSKKKETESAIYIFISDTGRPFVEMYSEIPEIIHMTEGRELVIPCRV
TSPNITVTLKKFPLDTLIPDGKRIIWDSRKGFIISNATYKEIGLLTCEATVNGHLYKTNYLTHRQTNTIIDVQISTPRPV
KLLRGHTLVLNCTATTPLNTRVQMTWSYPDEKNKRASVRRRIDQSNSHANIFYSVLTIDKMQNKDKGLYTCRVRSGPSFK
SVNTSVHIYDKAFITVKHRKQQVLETVAGKRSYRLSMKVKAFPSPEVVWLKDGLPATEKSARYLTRGYSLIIKDVTEEDA
GNYTILLSIKQSNVFKNLTATLIVNVKPQIYEKAVSSFPDPALYPLGSRQILTCTAYGIPQPTIKWFWHPCNHNHSEARC
DFCSNNEESFILDADSNMGNRIESITQRMAIIEGKNKMASTLVVADSRISGIYICIASNKVGTVGRNISFYITDVPNGFH
VNLEKMPTEGEDLKLSCTVNKFLYRDVTWILLRTVNNRTMHYSISKQKMAITKEHSITLNLTIMNVSLQDSGTYACRARN
VYTGEEILQKKEITIRDQEAPYLLRNLSDHTVAISSSTTLDCHANGVPEPQITWFKNNHKIQQEPGIILGPGSSTLFIER
VTEEDEGVYHCKATNQKGSVESSAYLTVQGTSDKPNLELITLTCTCVAATLFWLLLTLFIGGGSGGGSKSMSSMVSDTSC
TFPSSDGIFWKHWIQTKDGQCGSPLVSTRDGFIVGIHSASNFTNTNNYFTSVPKNFMELLTNQEAQQWVSGWRLNADSVL
WGGHKVFMVKPEEPFQPVKEATQLMNRRRRPGGGSENLYFQGPKKKRKVGGGSTSYPYDVPDYAGGSGSGGGSKPAFLSG
EQKKAIVDLLFKTNRKVTVKQLKEDYFKKIECFDSVEISGVEDRFNASLGTYHDLLKIIKDKDFLDNEENEDILEDIVLT
LTLFEDREMIEERLKTYAHLFDDKVMKQLKRRRYTGWGRLSRKLINGIRDKQSGKTILDFLKSDGFANRNFMQLIHDDSL
TFKEDIQKAQVSGQGDSLHEHIANLAGSPAIKKGILQTVKVVDELVKVMGRHKPENIVIEMARENQTTQKGQKNSRERMK
RIEEGIKELGSQILKEHPVENTQLQNEKLYLYYLQNGRDMYVDQELDINRLSDYDVDHIVPQSFLKDDSIDNKVLTRSDK
ARGKSDNVPSEEVVKKMKNYWRQLLNAKLITQRKFDNLTKAERGGLSELDKAGFIKRQLVETRQITKHVAQILDSRMNTK
YDENDKLIREVKVITLKSKLVSDFRKDFQFYKVREINNYHHAHDAYLNAVVGTALIKKYPKLESEFVYGDYKVYDVRKMI
AKSEQEIGKATAKYFFYSNIMNFFKTEITLANGEIRKRPLIETNGETGEIVWDKGRDFATVRKVLSMPQVNIVKKTEVQT
GGFSKESILPKRNSDKLIARKKDWDPKKYGGFDSPTVAYSVLVVAKVEKGKSKKLKSVKELLGITIMERSSFEKNPIDFL
EAKGYKEVKKDLIIKLPKYSLFELENGRKRMLASAGELQKGNELALPSKYVNFLYLASHYEKLKGSPEDNEQKQLFVEQH
KHYLDEIIEQISEFSKRVILADANLDKVLSAYNKHRDKPIREQAENIIHLFTLTNLGAPAAFKYFDTTIDRKRYTSTKEV
LDATLIHQSITGLYETRIDLSQLGGDSPKKKRKVEASGRADALDDFDLDMLGSDALDDFDLDMLGSDALDDFDLDMLGSD

ALDDFDLDMLINGTASGSG**EGRGSLLTCGDVEENPGP**VSKLMASNFTQFVLVDNGGTGDVTVAPSNFANGVAEWISSNSR
SQAYKVTCSVRQSSAQKRKYTIKVEVPKVATQTVGGVELPVAAWRSYLNMELTIPIFATNSDCELIVKAMQGLLKDGNPI
PSAIAANSGIYSA*GGGGSGGGGSGGGGS*GPKKKRKVAAAGS***PSGQISNQALALAPSSAPVLAQTMVPSSAMVPLAQPPAP***
APVLTPGPPQSLSAPVPKSTQAGEGTLSEALLHLQFDADEDLGALLGNSTDPGVFTDLASVDNSEFQQLLNQGVSMSHST
AEPMLMEYPEAITRLVTGSQRPPDPAPTPLGTSGLPNGLSGDEDFSSIADMDFSALLSQISSSGQGGGGS**GFSVDTSALL**
DLFSPSVTVPDMSLPDLDSSLASIQELLSPQEPPRPPEAENSSPDSGKQLVHYTAQPLFLLDPGSVDTGSNDLPVLFELG
EGSYFSEGDGFAEDPTISLLTGSEPPKAKDPTVS*

NN VEGFR1 leader peptide, the extracellular domains and transmembrane domain
NN C-TEV
NN efficient TCS; tobacco etch virus (TEV) protease cleavage sequence
NN NLS, SV40 nuclear localisation sequence
NN HA, hemagglutinin A epitope
NN dCas9(C), C-terminal moiety of human codon–optimized *Streptococcus pyogenes* Cas9
NN VP64, transcriptional activator domain
NN T2A, 2A self-cleaving peptide from thosea asigna virus
NN MCP (MS2 protein), MS2 bacteriophage coat protein
NN P65, activation domain from human NF-kB trans-activating subunit p65
NN HSF1, activation domains from human heat-shock factor 1

dCas9(N)-synVEGFR-2 used in *pVEGFR2_TEV(N)_NES_TCS(Q'L)_HA-dCas9(N)_P2A_Puro-WPRE*

MQSKVLLAVALWLCVETRAASVGLPSVSLDLPRLSIQKDILTIKANTTLQITCRGQRDLDWLWPNNQSGSEQRVEVTECS
DGLFCKTLTIPKVIGNDTGAYKCFYRETDLASVIYVYVQDYRSPFIASVSDQHGVVYITENKNKTVVIPCLGSISNLNVS
LCARYPEKRFVPDGNRISWDSKKGFTIPSYMISYAGMVFCEAKINDESYQSIMYIVVVVGYRIYDVVLSPSHGIELSVGE
KLVLNCTARTELNVGIDFNWEYPSSKHQHKKLVNRDLKTQSGSEMKKFLSTLTIDGITRSDQGLYTCAASSGLMTKKNST
FVRVHEKPFVAFGSGMESLVEATVGERVRIPAKYLGYPPPEIKWYKNGIPLESNHTIKAGHVLTIMEVSERDTGNYTVIL
TNPISKEKQSHVVSLVVYVPPQIGEKSLISPVDSYQYGTTQTLTCTVYAIPPPHHIHWYWQLEEECANEPSQAVSVTNPY
PCEEWRSVEDFQGGNKIEVNKNQFALIEGKNKTVSTLVIQAANVSALYKCEAVNKVGRGERVISFHVTRGPEITLQPDMQ
PTEQESVSLWCTADRSTFENLTWYKLGPQPLPIHVGELPTPVCKNLDTLWKLNATMFSNSTNDILIMELKNASLQDQGDY
VCLAQDRKTKKRHCVVRQLTVLERVAPTITGNLENQTTSIGESIEVSCTASGNPPPQIMWFKDNETLVEDSGIVLKDGNR
NLTIRRVRKEDEGLYTCQACSVLGCAKVEAFFIIEGAQEKTNLEIIILVGTAVIAMFFWLLLVIIGGGSGGGS*GESLFKG*
PRDYNPISSTICHLTNESDGHTTSLYGIGFGPFIITNKHLFRRNNGTLLVQSLHGVFKVKNTTTLQQHLIDGRDMIIRM
*PKDFPPFPQKLKFREPQREERICLVTTNFQT*GGGSLDLASLILGKLG*ENLYFQL*GGGSTS*YPYDVPDYA*GGSGSDKKYSI
GLAIGTNSVGWAVITDEYKVPSKKFKVLGNTDRHSIKKNLIGALLFDSGETAEATRLKRTARRRYTRRKNRICYLQEIFS
NEMAKVDDSFFHRLEESFLVEEDKKHERHPIFGNIVDEVAYHEKYPTIYHLRKKLVDSTDKADLRLIYLALAHMIKFRGH
FLIEGDLNPDNSDVDKLFIQLVQTYNQLFEENPINASGVDAKAILSARLSKSRRLENLIAQLPGEKKNGLFGNLIALSLG
LTPNFKSNFDLAEDAKLQLSKDTYDDDLDNLLAQIGDQYADLFLAAKNLSDAILLSDILRVNTEITKAPLSASMIKRYDE
HHQDLTLLKALVRQQLPEKYKEIFFDQSKNGYAGYIDGGASQEEFYKFIKPILEKMDGTEELLVKLNREDLLRKQRTFDN
GSIPHQIHLGELHAILRRQEDFYPFLKDNREKIEKILTFRIPYYVGPLARGNSRFAWMTRKSEETITPWNFEEVVDKGAS
AQSFIERMTNFDKNLPNEKVLPKHSLLYEYFTVYNELTKVKYVTEGMRGGGGSGTGS**GATNFSLLKQAGDVEENPGP**EF*M*
TEYKPTVRLATRDDVPRAVRTLAAAFADYPATRHTVDPDRHIERVTELQELFLTRVGLDIGKVWVADDGAAVAVWTTPES
VEAGAVFAEIGPRMAELSGSRLAAQQQMEGLLAPHRPKEPAWFLATVGVSPDHQGKGLGSAVVLPGVEAAERAGVPAFLE
*TSAPRNLPFYERLGFTVTADVEVPEGPRTWCMTRKG**

NN VEGFR2 leader peptide, the extracellular domains and transmembrane domain
NN N-TEV
NN NES, nuclear export signal form human protein tyrosine kinase 2
NN inefficient TCS (Q'L); tobacco etch virus (TEV) protease cleavage sequence
NN HA, hemagglutinin A epitope
NN dCas9(N), N-terminal moiety of human codon–optimized *Streptococcus pyogenes* Cas9
NN P2A, 2A self-cleaving peptide from porcine teschovirus-1
NN Puromycin resistance protein

dCas9(N)-synVEGFR2^{RI}

MQSKVLLAVALWLCVETRAASVGLPSVSLDLPRLSIQKDILTIKANTTLQITCRGQRDLDWLWPNNQSGSEQRVEVTECS
DGLFCKTLTIPKVIGNDTGAYKCFYRETDLASVIYVYVQDYRSPFIASVSDQHGVVYITENKNKTVVIPCLGSISNLNVS
LCARYPEKRFVPDGNRISWDSKKGFTIPSYMISYAGMVFCEAKINDESYQSIMYIVVVVGYRIYDVVLSPSHGIELSVGE
KLVLNCTARTELNVGIDFNWEYPSSKHQHKKLVNRDLKTQSGSEMKKFLSTLTIDGITRSDQGLYTCAASSGLMTKKNST
FVRVHEKPFVAFGSGMESLVEATVGERVRIPAKYLGYPPPEIKWYKNGIPLESNHTIKAGHVLTIMEVSERDTGNYTVIL
TNPISKEKQSHVVSLVVYVPPQIGEKSLISPVDSYQYGTTQTLTCTVYAIPPPHHIHWYWQLEEECANEPSQAVSVTNPY
PCEEWRSVEDFQGGNKIEVNKNQFALIEGKNKTVSTLVIQAANVSALYKCEAVNKVGRGERVISFHVTRGPEITLQPDMQ
PTEQESVSLWCTADRSTFENLTWYKLGPQPLPIHVGELPTPVCKNLDTLWKLNATMFSNSTNDILIMELKNASLQDQGDY

VCLAQDRKTKKRHCVVRQLTVLERVAPTITGNLENQTTSIGESIEVSCTASGNPPPQIMWFKDNETLVEDSGIVLKDGNR
NLTIRRVRKEDEGLYTCQACSVLGCAKVEAFFIIEGAQEKTNLEIIILVGTAVIAMFFWLLLVIIGGGSGGGS*GESLFKG*
PRDYNPISSTICHLTNESDGHTTSLYGIGFGPFIITNKHLFRRNNGTLLVQSLHGVFKVKNTTTLQQHLIDGRDMIIIRM
*PKDFPPFPQKLKFREPQREERICLVTTNFQT*GGGSLDLASLILGKLG*ENLYFQL*GGGSTSGVQVETISPGDGRTFPKRGQ
TCVVHYTGMLEDGKKFDSSRDRNKPFKFMLGKQEVIRGWEEGVAQMSVGQRAKLTISPDYAYGATGHPGIIPPHATLVFD
VELLKLETS*YPYDVPDYA*GGSGSDKKYSIGLAIGTNSVGWAVITDEYKVPSKKFKVLGNTDRHSIKKNLIGALLFDSGET
AEATRLKRTARRRYTRRKNRICYLQEIFSNEMAKVDDSFFHRLEESFLVEEDKKHERHPIFGNIVDEVAYHEKYPTIYHL
RKKLVDSTDKADLRLIYLALAHMIKFRGHFLIEGDLNPDNSDVDKLFIQLVQTYNQLFEENPINASGVDAKAILSARLSK
SRRLENLIAQLPGEKKNGLFGNLIALSLGLTPNFKSNFDLAEDAKLQLSKDTYDDDLDNLLAQIGDQYADLFLAAKNLSD
AILLSDILRVNTEITKAPLSASMIKRYDEHHQDLTLLKALVRQQLPEKYKEIFFDQSKNGYAGYIDGGASQEEFYKFIKP
ILEKMDGTEELLVKLNREDLLRKQRTFDNGSIPHQIHLGELHAILRRQEDFYPFLKDNREKIEKILTFRIPYYVGPLARG
NSRFAWMTRKSEETITPWNFEEVVDKGASAQSFIERMTNFDKNLPNEKVLPKHSLLYEYFTVYNELTKVKYVTEGMRGGG
GSGTGS**GATNFSLLKQAGDVEENPGP**EF*MTEYKPTVRLATRDDVPRAVRTLAAAFADYPATRHTVDPDRHIERVTELQEL*
FLTRVGLDIGKVWVADDGAAVAVWTTPESVEAGAVFAEIGPRMAELSGSRLAAQQQMEGLLAPHRPKEPAWFLATVGVSP
*DHQGKGLGSAVVLPGVEAAERAGVPAFLETSAPRNLPFYERLGFTVTADVEVPEGPRTWCMTRKG**

NN VEGFR2 leader peptide, the extracellular domains and transmembrane domain
NN N-TEV
NN NES, nuclear export signal form human protein tyrosine kinase 2
NN inefficient TCS (Q'L); tobacco etch virus (TEV) protease cleavage sequence
NN FKBP12, FK506 binding protein 12
NN HA, hemagglutinin A epitope
NN dCas9(N), N-terminal moiety of human codon–optimized *Streptococcus pyogenes* Cas9
NN P2A, 2A self-cleaving peptide from porcine teschovirus-1
NN Puromycin resistance protein

dCas9(C)-synBDKRB2 used in *pBDKRB2_TCS (Q'G)_NLS-HA-dCas9(C)-VP64_T2A_MCP-P65-HSF1*

MKTIIALSYIFCLVFADYKDDDDASID*MFSPWKISMFLSVREDSVPTTASFSADMLNVTLQGPTLNGTFAQSKCPQVEWL*
GWLNTIQPPFLWVLFVLATLENIFVLSVFCLHKSSCTVAEIYLGNLAAADLILACGLPFWAITISNNFDWLFGETLCRVV
NAIISMNLYSSICFLMLVSIDRYLALVKTMSMGRMRGVRWAKLYSLVIWGCTLLLSSPMLVFRTMKEYSDEGHNVTACVI
SYPSLIWEVFTNMLLNVVGFLLPLSVITFCTMQIMQVLRNNEMQKFKEIQTERRATVLVLVVLLLFIICWLPFQISTFLD
TLHRLGILSSCQDERIIDVITQIASFMAYSNSCLNPLVYVIVGKRFRKKSWEVYQGVCQKGGCRSEPIQMENSMGTLRTS
*ISVERQIHKLQDWAGSRQ*IDTGGRTPPSLGPQDESCTTASSSLAKDTSSTG*ENLYFQG*PKKKRKVGGGSTS*YPYDVPDYA*
GGSGSGGGSKPAFLSGEQKKAIVDLLFKTNRKVTVKQLKEDYFKKIECFDSVEISGVEDRFNASLGTYHDLLKIIKDKDF
LDNEENEDILEDIVLTLTLFEDREMIEERLKTYAHLFDDKVMKQLKRRRYTGWGRLSRKLINGIRDKQSGKTILDFLKSD
GFANRNFMQLIHDDSLTFKEDIQKAQVSGQGDSLHEHIANLAGSPAIKKGILQTVKVVDELVKVMGRHKPENIVIEMARE
NQTTQKGQKNSRERMKRIEEGIKELGSQILKEHPVENTQLQNEKLYLYYLQNGRDMYVDQELDINRLSDYDVDHIVPQSF
LKDDSIDNKVLTRSDKARGKSDNVPSEEVVKKMKNYWRQLLNAKLITQRKFDNLTKAERGGLSELDKAGFIKRQLVETRQ
ITKHVAQILDSRMNTKYDENDKLIREVKVITLKSKLVSDFRKDFQFYKVREINNYHHAHDAYLNAVVGTALIKKYPKLES
EFVYGDYKVYDVRKMIAKSEQEIGKATAKYFFYSNIMNFFKTEITLANGEIRKRPLIETNGETGEIVWDKGRDFATVRKV
LSMPQVNIVKKTEVQTGGFSKESILPKRNSDKLIARKKDWDPKKYGGFDSPTVAYSVLVVAKVEKGKSKKLKSVKELLGI
TIMERSSFEKNPIDFLEAKGYKEVKKDLIIKLPKYSLFELENGRKRMLASAGELQKGNELALPSKYVNFLYLASHYEKLK
GSPEDNEQKQLFVEQHKHYLDEIIEQISEFSKRVILADANLDKVLSAYNKHRDKPIREQAENIIHLFTLTNLGAPAAFKY
FDTTIDRKRYTSTKEVLDATLIHQSITGLYETRIDLSQLGGDSPKKKRKVEAS**GRADALDDFDLDMLGSDALDDFDLDML**
GSDALDDFDLDMLGSDALDDFDLDMLINGTASGSG**EGRGSLLTCGDVEENPGP**VSKLMASNFTQFVLVDNGGTGDVTVAP
SNFANGVAEWISSNSRSQAYKVTCSVRQSSAQKRKYTIKVEVPKVATQTVGGVELPVAAWRSYLNMELTIPIFATNSDCE
LIVKAMQGLLKDGNPIPSAIAANSGIYSA*GGGGSGGGGSGGGGS*GPKKKRKVAAAGS***PSGQISNQALALAPSSAPVLAQT***
MVPSSAMVPLAQPPAPAPVLTPGPPQSLSAPVPKSTQAGEGTLSEALLHLQFDADEDLGALLGNSTDPGVFTDLASVDNS
EFQQLLNQGVSMSHSTAEPMLMEYPEAITRLVTGSQRPPDPAPTPLGTSGLPNGLSGDEDFSSIADMDFSALLSQISSSG
QGGGGS**GFSVDTSALLDLFSPSVTVPDMSLPDLDSSLASIQELLSPQEPPRPPEAENSSPDSGKQLVHYTAQPLFLLDPG**
SVDTGSNDLPVLFELGEGSYFSEGDGFAEDPTISLLTGSEPPKAKDPTVS*

NN membrane localisation signal sequence and Flag epitope tag
NN BDKRB2 coding sequence
NN V_2-tail
NN efficient TCS; tobacco etch virus (TEV) protease cleavage sequence
NN NLS, SV40 nuclear localisation sequence
NN HA, hemagglutinin A epitope
NN dCas9(C), C-terminal moiety of human codon–optimized *Streptococcus pyogenes* Cas9
NN VP64, transcriptional activator domain
NN T2A, 2A self-cleaving peptide from thosea asigna virus
NN MCP (MS2 protein), MS2 bacteriophage coat protein
NN P65, activation domain from human NF-kB trans-activating subunit p65
NN HSF1, activation domains from human heat-shock factor 1

Note: The same modular scaffold was used to create dCas9(C)-synAVPR2, dCas9(C)-synCXCR4, dCas9(C)-synLPAR1 and dCas9(C)-synT1R3 (see Supplemental Experimental Procedures).

dCas9(N)-synBDKRB2 used in *pBDKRB2_TCS(Q'L)_HA-dCas9(N)_P2A-Puro-WPRE*

MKTIIALSYIFCLVFADYKDDDDASID*MFSPWKISMFLSVREDSVPTTASFSADMLNVTLQGPTLNGTFAQSKCPQVEWL*
GWLNTIQPPFLWVLFVLATLENIFVLSVFCLHKSSCTVAEIYLGNLAAADLILACGLPFWAITISNNFDWLFGETLCRVV
NAIISMNLYSSICFLMLVSIDRYLALVKTMSMGRMRGVRWAKLYSLVIWGCTLLLSSPMLVFRTMKEYSDEGHNVTACVI
SYPSLIWEVFTNMLLNVVGFLLPLSVITFCTMQIMQVLRNNEMQKFKEIQTERRATVLVLVVLLLFIICWLPFQISTFLD
TLHRLGILSSCQDERIIDVITQIASFMAYSNSCLNPLVYVIVGKRFRKKSWEVYQGVCQKGGCRSEPIQMENSMGTLRTS
*ISVERQIHKLQDWAGSRQ*IDTGGRTPPSLGPQDESCTTASSSLAKDTSSTG*ENLYFQL*TS*YPYDVPDYA*GGSGSDKKYSI
GLAIGTNSVGWAVITDEYKVPSKKFKVLGNTDRHSIKKNLIGALLFDSGETAEATRLKRTARRRYTRRKNRICYLQEIFS
NEMAKVDDSFFHRLEESFLVEEDKKHERHPIFGNIVDEVAYHEKYPTIYHLRKKLVDSTDKADLRLIYLALAHMIKFRGH
FLIEGDLNPDNSDVDKLFIQLVQTYNQLFEENPINASGVDAKAILSARLSKSRRLENLIAQLPGEKKNGLFGNLIALSLG
LTPNFKSNFDLAEDAKLQLSKDTYDDDLDNLLAQIGDQYADLFLAAKNLSDAILLSDILRVNTEITKAPLSASMIKRYDE
HHQDLTLLKALVRQQLPEKYKEIFFDQSKNGYAGYIDGGASQEEFYKFIKPILEKMDGTEELLVKLNREDLLRKQRTFDN
GSIPHQIHLGELHAILRRQEDFYPFLKDNREKIEKILTFRIPYYVGPLARGNSRFAWMTRKSEETITPWNFEEVVDKGAS
AQSFIERMTNFDKNLPNEKVLPKHSLLYEYFTVYNELTKVKYVTEGMRGGGGSGTGS**GATNFSLLKQAGDVEENPGP**EF*M*
TEYKPTVRLATRDDVPRAVRTLAAAFADYPATRHTVDPDRHIERVTELQELFLTRVGLDIGKVWVADDGAAVAVWTTPES
VEAGAVFAEIGPRMAELSGSRLAAQQQMEGLLAPHRPKEPAWFLATVGVSPDHQGKGLGSAVVLPGVEAAERAGVPAFLE
*TSAPRNLPFYERLGFTVTADVEVPEGPRTWCMTRKG**

NN membrane localisation signal sequence and Flag epitope tag
NN BDKRB2 coding sequence
NN V_2-tail
NN inefficient TCS (Q'L); tobacco etch virus (TEV) protease cleavage sequence
NN HA, hemagglutinin A epitope
NN dCas9(N), N-terminal moiety of human codon–optimized *Streptococcus pyogenes* Cas9
NN P2A, 2A self-cleaving peptide from porcine teschovirus-1
NN Puromycin resistance protein

Note: The same modular scaffold was used to create dCas9(N)-synAVPR2, dCas9(N)-synCXCR4, dCas9(N)-synLPAR1 and dCas9(N)-synT1R3 (see Supplemental Experimental Procedures).
